# When does risk become residual? A systematic review of research on flood risk management in West Africa

**DOI:** 10.1007/s10113-021-01826-7

**Published:** 2021-08-25

**Authors:** Simon Wagner, Maxime Souvignet, Yvonne Walz, Kehinde Balogun, Kossi Komi, Sönke Kreft, Jakob Rhyner

**Affiliations:** 1 United Nations University – Institute for Environment and Human Security (UNU-EHS), UN Campus Platz der Vereinten Nationen 1, 53113 Bonn, Germany; 2 Agricultural Faculty, University of Bonn, Meckenheimer Allee 174, 53115 Bonn, Germany; 3 Laboratoire de Recherche Sur Les Espaces, Les Echanges Et La Sécurité Humaine, Département de Géographie, Université de Lomé, BP: 1515, Lomé, Togo

**Keywords:** Flood, Residual risk, Risk management, West Africa, Systematic review

## Abstract

Flood events in West Africa have devastating impacts on the lives of people. Additionally, developments such as climate change, settlement expansion into flood-prone areas, and modification of rivers are expected to increase flood risk in the future. Policy documents have issued calls for conducting local risk assessments and understanding disaster risk in diverse aspects, leading to an increase in such research. Similarly, in a shift from flood protection to flood risk management, the consideration of various dimensions of flood risk, the necessity of addressing flood risk through an integrated strategy containing structural and non-structural measures, and the presence of residual risk are critical perspectives raised. However, the notion of “residual risk” remains yet to be taken up in flood risk management-related academic literature. This systematic review seeks to approach the notion of residual risk by reviewing information on flood impacts, common measures, and recommendations in academic literature. The review reveals various dimensions of impacts from residual flood risk aside from material damage, in particular, health impacts and economic losses. Infrastructural measures were a dominant category of measures before and after flood events and in recommendations, despite their shortcomings. Also, spatial planning interventions, a more participatory and inclusive governance approach, including local knowledge, sensitisation, and early warning systems, were deemed critical. In the absence of widespread access to insurance schemes, support from social networks after flood events emerged as the most frequent measure. This finding calls for in-depth assessments of those networks and research on potential complementary formal risk transfer mechanisms.

**Supplementary Information:**

The online version contains supplementary material available at 10.1007/s10113-021-01826-7.

## Introduction

Flood events in West Africa have inflicted devastating impacts on the lives of its inhabitants (Badou et al. 2019). Region-wide flood events, such as in 2007 (UN OCHA 2007), 2009 (UN OCHA 2009), 2010 (UN OCHA 2010), 2012 (UN OCHA 2012), 2016 (UN OCHA 2016), or most recently in 2020 (ERCC 2020), illustrate they are reoccurring more frequently, and with high severity in many places, causing large-scale loss and damage. The Emergency Events Database (EM-Dat), which records essential disaster data on a global scale, identifies 249 large-scale flood events (> 10 fatalities or 100 affected people), which caused approximately 3800 deaths and affecting about 25 million people from 1991 to 2019 in the Economic Community of West African States (ECOWAS) (EM-Dat 2020). ECOWAS member states include Benin, Burkina Faso, Ivory Coast, The Gambia, Ghana, Guinea, Guinea-Bissau, Liberia, Mali, Niger, Nigeria, Senegal, Sierra Leone, Cape Verde, and Togo (ECOWAS 2020). Furthermore, despite uncertainties in several precipitation indices (Dosio et al. 2019), Global Climate Models (GCMs) and Regional Climate Models (RCMs) indicate shorter, more intense, and later rainy seasons for West Africa due to climate change (Vizy and Cook 2012; Dunning and Black 2018; Dosio et al. 2019). This trend is expected to lead to an increase in harmful flood and drought events in the region (Akinsanola and Zhou 2019). Moreover, human activity, such as dam construction, alters natural river regimes (Mahe et al. 2013), whilst intensive urban expansion is projected to continue in flood exposed areas such as the Niger river and low-elevation coastal zones (LECZ) along the Gulf of Guinea up to 2030 (Güneralp et al. 2015).

In research as well as policy-making, there has been a growing awareness for the need to shift from a flood protection paradigm to flood risk management (FRM) (Hartmann and Albrecht 2014; Evers et al. 2016; Thomas and Knüppe 2016; Roos et al. 2017). Whilst in the conventional flood protection paradigm, floods are usually addressed in a top-down manner by centrally implemented structural measures; an FRM approach calls for an integrated and synergetic combination of structural and non-structural measures implemented by various actors in a polycentral and participatory manner (Grabs et al. 2007; WMO 2009; Sayers et al. 2013; Challies et al. 2016; Milman et al. 2018). Contrary to conventional flood protection approaches, FRM also led to the perspective that flood risk can seldomly be reduced entirely, thus requiring strategies to address the residual risk that remains unaddressed despite risk-reducing measures being in place or their potential failure (Plate 2002; Ludy and Kondolf 2012; Arrighi et al. 2018). Similarly, according to current perspectives in the field of Disaster Risk Reduction (DRR), residual risk is termed as “the disaster risk that remains in unmanaged form, even when effective disaster risk reduction measures are in place, and for which emergency response and recovery capacities must be maintained” (UNDRR 2020a, online). Therefore, “the presence of residual risk implies a continuing need to develop and support effective capacities for emergency services, preparedness, response and recovery, together with socioeconomic policies such as safety nets and risk transfer mechanisms, as part of a holistic approach” (UNDRR 2020a, online).

In addition, FRM seeks an expansion of risk dimensions to encompass not only the possibility of material damage but also health impacts, economic damages, the destruction of cultural heritage or impaired livelihood opportunities, and ensuing poverty (EU 2007; WMO 2009). The need for a broader and more thorough understanding of disaster risk as a basis for achieving DRR has also been underscored in the realm of policy. For example, in 1989, the United Nations proclaimed the decade of 1990–2000 as the “International Decade for Natural Disaster Reduction” to enhance international cooperation on the topic (UN 1989). Moreover, the Hyogo Framework for Action 2005–2015 (HFA) already called for local risk assessments and to effectively integrate disaster risk considerations into policies, planning, and programming (UN 2005). Also, with its first priority, the ensuing Sendai Framework for Disaster Risk Reduction 2015–2030 (SFDRR) emphasises the importance of understanding disaster risk in all its dimensions (such as vulnerability, capacity, exposure, and hazard) as well as their interconnected impacts to inform disaster risk management meaningfully (UN 2015). Those developments have led to an increased number of publications discussing local flood impacts and efforts of FRM within the academic literature, also for the West African region. However, those publications are mainly case studies and thus primarily provide context-specific information on a local level.

Previous review studies on academic literature relating to FRM in West Africa have not yet summarised works for the entire region with a systematic review approach. On the regional scale, work focussing on such literature includes a review of gaps and challenges of FRM that has been carried out in four selected coastal West African cities (Ouikotan et al. 2017). However, besides considering a limited number of coastal cities, it did not apply a systematic review approach. Similarly, Badou et al. (2019) have carried out a literature review that summarised flood statistics, triggers of floods, solutions for prevention and mitigation of flood effects as mentioned by research, and future research priorities. Even though it is based on academic case studies, it does not offer a systematic approach to the research synthesis. Moreover, FRM-related review studies in the West African region have often focussed on one country or city. Also, they are either occupied with Nigeria or Ghana. On the one hand, for Nigeria, there are reviews on the impact of floods on Nigeria’s achievement of the sustainable development goals (Echendu 2020), on sustainable FRM-practices in flood-prone areas of Nigeria (Cirella and Iyalomhe 2018), on the challenges and opportunities of FRM in Nigeria (Oladokun and Proverbs 2016), and on the National Disaster Management Framework of Nigeria (Olanrewaju et al. 2019). For the city of Lagos in Nigeria, review papers examined the FRM practices of public and private actors (Adelekan 2016) and factors relating to the flood hazard, exposure and vulnerability, and challenges to reducing them (Nkwunonwo et al. 2016). On the other hand, for Ghana, there are reviews on current flood risk management practices as well as gaps and opportunities for improving resilience (Almoradie et al. 2020) and on emerging trends in FRM in the country (Ahadzie and Proverbs 2011). Of those reviews, only a few followed a systematic review approach. Furthermore, none of them explicitly considered the aspect of residual risk and how its resulting impacts are addressed. Therefore, applying such a review approach to all West African countries will enable a broader discussion of trends in FRM at the regional level.

**Fig. 1:**
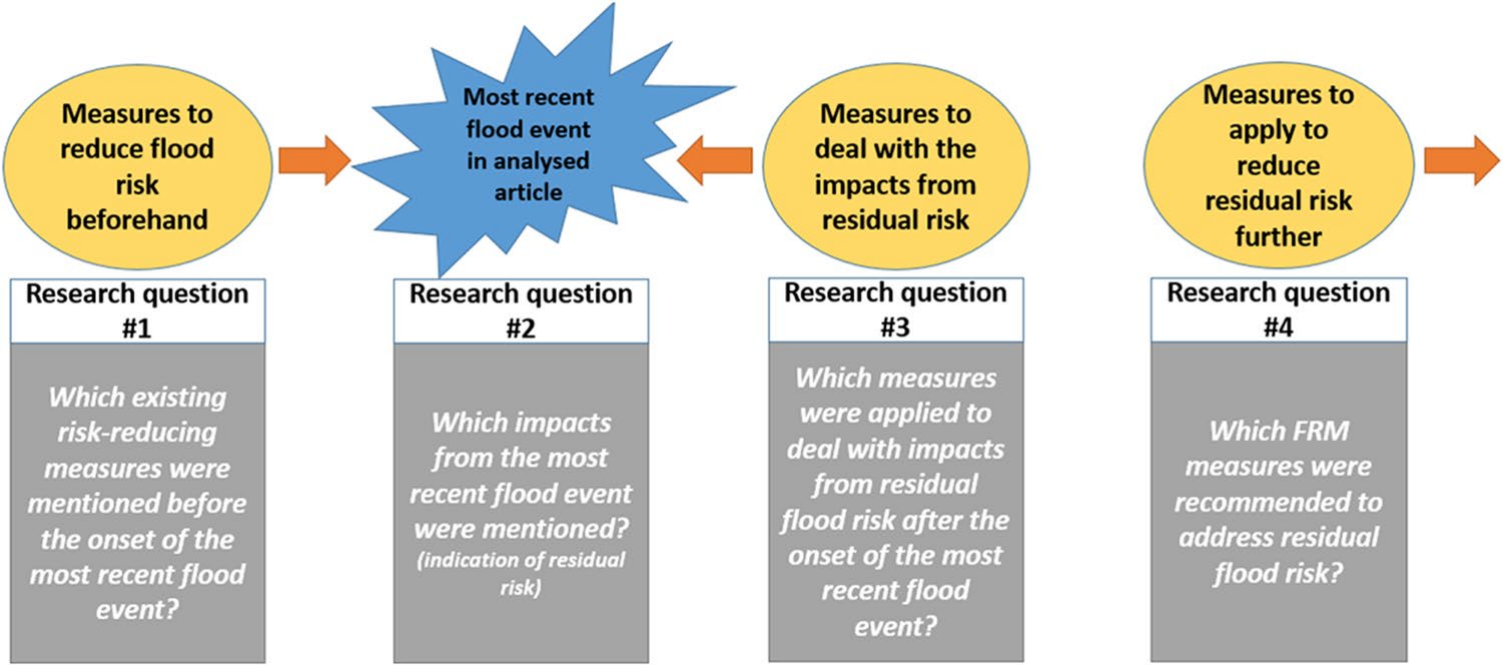
Analytical approach of the review paper and visualisation of research questions

The aim of this review is to better understand the role of residual risk in FRM-related research for the region of West Africa. To achieve this, the article provides a systematic review of academic literature (journal articles and book chapters) and the contextual information it provides for FRM-related aspects in the region of West Africa. The analytical approach of this paper and its research questions to collect data on FRM measures and residual risk is summarised in Fig. 1, drawing upon the perspectives of FRM and DRR mentioned above. This review’s approach is to use the onset of the most recent flood event contained within the case study as a point of reference, to determine whether risk remained unmanaged or not. Thus, this review first analyses those FRM measures that have been applied before the onset of the most recent flood event, as reported in the case study. Second, the analysis focusses on the observed flood impacts as evidence-based indications of residual flood risk that materialised despite previous risk-reducing measures being implemented. Third, measures that have been applied after the onset of the most recent flood event to deal with the impacts of residual flood risk are analysed. Finally, recommendations produced as part of research to further address residual flood risk will be summarised in this review.

## Method

**Fig. 2:**
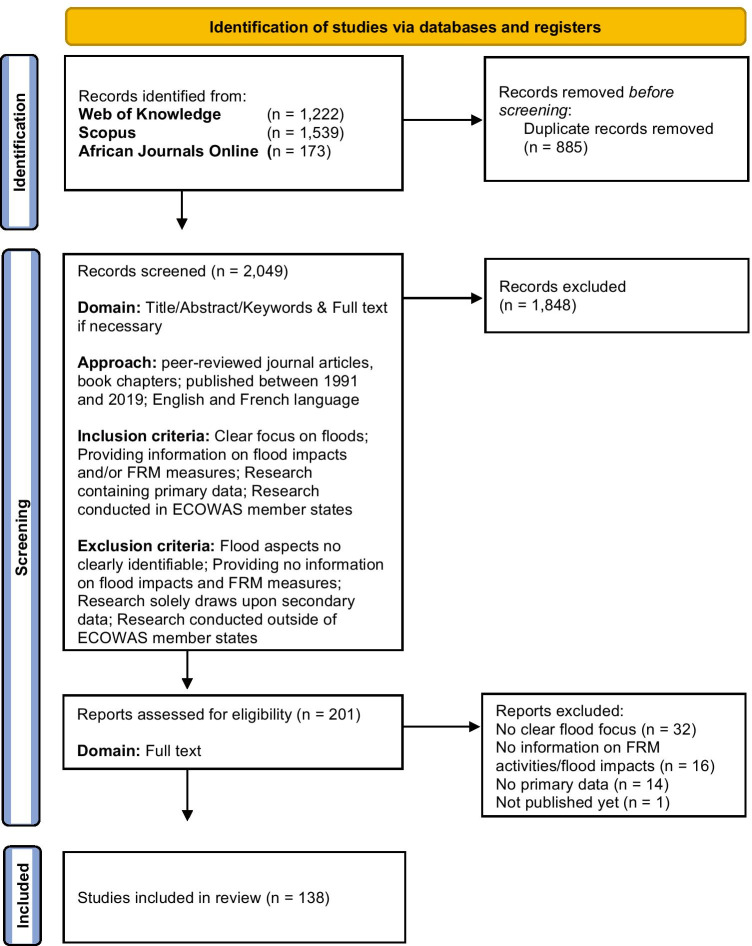
Flow chart of review procedure ( adapted from Page et al. 2021)

In environmental sciences, systematic reviews are increasingly carried out in research relating to climate change adaptation (Berrang-Ford et al. 2011; Ford et al. 2014, 2016; Lesnikowski et al. 2015; Epule et al. 2017; Biesbroek et al. 2018; Shaffril et al. 2018; Owen 2020), drought risk (Kamara et al. 2018; Hagenlocher et al. 2019), and to FRM (Wellens et al. 2013; Abbas et al. 2016; Nordbeck et al. 2019; Carrick et al. 2019) due to their ability to provide a comprehensive summary of existing trends and foci in academic and/or grey literature. However, the variation in methodological approaches and the varying levels of transparency have been pointed out and were met with a set of proposed components by Berrang-Ford et al. (2015) for the standardisation of such research concerning the research questions/aim, data source, and document selection, and analysis and presentation of results. This study is seeking to address each of those aspects as a guide for enhanced transparency in this review paper. Furthermore, the article draws upon guidance from Siddaway et al. (2019) and Mengist et al. (2020) on the procedure of carrying out this systematic review, which is outlined in the section. Also, the article illustrates the review process in the form of a flow chart (Fig. 2) as recommended by the Preferred Reporting Items for Systematic Reviews and Meta-Analyses (PRISMA) statement, which formulates a minimum set of items for reporting the review procedure (Page et al. 2021).

Documents that were written in either English or French were searched for using sets of relevant English and French search terms (Annex 1). The keywords were selected in those languages since they are the most prevalent official languages in the ECOWAS region (with the exception of Guinea-Bissau and Cape Verde). Research areas in selected documents were mapped to illustrate a potential reporting bias in the geographical representation of West African countries in the final data set. Research published from 1991 onward up to 2019 was selected because the earliest large-scale flood event within the UN’s “International Decade for Natural Disaster Reduction” (1990–2000) in ECOWAS states listed on the EM-Dat database occurred in 1991 (EM-Dat 2020). The final set of search terms was selected in an iterative process by seeking additional keywords identified in relevant articles that were in previously identified documents. The saturation point was deemed to be reached when several newly added search terms were only adding a small single-digit number to the number of articles obtained by the query. The final set of terms was searched on 29th July 2020.

As outlined in Fig. 2, relevant literature was searched for in Web of Knowledge and Scopus because they are the most extensive databases for peer-reviewed research. Additionally, African Journals Online (AJOL) was included as a database because it contained additional relevant research from local research institutions that mainly were not listed in the other two databases. However, the authors are aware that additional relevant research might be published in other databases as those considered for this review. After the initial search yielded 2934 documents, 885 duplicates were removed, which resulted in a list of 2049 unique documents. Original research articles, in the form of peer-reviewed articles and book chapters containing primary data from field-based research, were selected as document types for this review. The explicit explanation of the primary data collection process was taken as a quality criterion for the inclusion of a document into the review.

The retrieved documents were screened in three rounds of review. The first round of screening was done by the primary author, who assessed the title, abstract, and keywords of each article, indicating their relevance by stating “yes”, “no”, or “perhaps”. Similarly, in the second round of screening, the entire list of articles was assessed independently by a team of eight reviewers to minimise personal selection bias, of which each member received a share of the entire set of articles. The team of reviewers then also indicated the relevance of each article by stating “yes”, “no”, or “perhaps”, without seeing the results of the first round of screening. The purpose of the third review round was to arbitrate judgments in case the first and the second rounds of review differed in their judgment, or if both parties submitted “perhaps”. The final reviewer indicated “yes” or “no” to make the final judgment based on the title, abstract keywords, and the full article if necessary. All reviewers assessed the relevance of articles based on the same inclusion and exclusion criteria (Fig. 2). Documents were included if they unambiguously focussed on floods but excluded if they combined information about flood impacts or FRM measures with other hazards or with climate change in an inseparable way. Also, studies were excluded that focussed merely on assessing the physical flood hazard but provided no information on the research questions. Contrarily, those that contained information on impacts and responses (flood impacts from FRM measures before and after the most recent flood event or recommended measures to reduce residual flood risk further) were included. Finally, only research that contained primary data and that was carried out in the selected West African countries of interest was included. Selected West African countries are the member states of ECOWAS, namely Benin, Togo, Senegal, The Gambia, Guinea, Guinea-Bissau, Mali, Ivory Coast, Sierra Leone, Burkina Faso, Niger, Nigeria, Ghana, Liberia, and Cape Verde. Research that was purely based on secondary data or carried out outside of the countries of interest was excluded.

The process of screening by the reviewers led to a selection of 201 documents, which were read in their entirety to decide about their eligibility. The coding of information relating to the research questions was done by three reviewers, including the main author, using the software MAXQDA 2020. The reviewer team chose the software because of being able to easily exchange and merge project documents and its easy-to-operate user interface for coding text (VERBI 2021). Also, Excel sheets summarising each code can be exported and used to visualise and analyse the data as done for this review. To minimise bias in coding articles and deciding on their eligibility, the main author and the two other reviewers went through all 201 documents twice. If an impact or measure was mentioned to occur, or to be carried out, in a document, it was captured through open coding in MAXQDA. In this process, categories for impacts and measures emerged through continuously grouping and regrouping the results (Table 1). The information on impacts and measures are summarised by using descriptive statistics in this review. In addition, the working definitions for the categories of impacts and measures, as well as a comprehensive overview of the composition of each category of measures, can be found in Annex 8, 9, 10, and 11. Coded measures and impacts are counted once per document if they appear in the case study. This approach was chosen because the main research aim is to showcase the range of the composition of applied or recommended measures in FRM and the dimension of impacts in the case studies. It should also be made clear that one single document can have research areas in multiple countries. By reading the documents in their entirety during the coding process, 138 were finally included (Annex 2) and considered to be relevant for this review, also based on whether each document met the inclusion and exclusion criteria whilst being read in full-length (Fig. 2). In this process, 32 documents were excluded for not focussing on floods clearly enough, 16 for not providing enough information on FRM measures or flood impacts, 14 for not containing primary data, and 1 for not being published yet.

**Table 1:** List of indicators guiding data collection

Indicator	Categories	Sub-categories
Country	Nigeria, Ghana, Senegal, Benin, Niger, Burkina Faso, Togo, Ivory Coast, Cape Verde, The Gambia, Sierra Leone, Mali, Guinea-Bissau, Liberia, Guinea	
Geographical area	Urban, coastal zone, rural, peri-urban, delta region	
Types of floods	Pluvial flood, fluvial flood, coastal flood, groundwater flood	
Methods used for data collection	Surveys, qualitative interviews (semi-structured, in-depth, key informant), field observations, focus groups, photography/photo elicitation, workshops, stakeholder meetings, transect walks, collective mapping	
FRM measures (before and after the onset of the most recent flood event and recommended)	Infrastructural	Drainage construction, flood defense structures, elevation of buildings or infrastructure, dams/dikes, land filling (sand, stone, waste, etc.), dredging river channels/channelisation, riverbank reinforcement/embankments, water storage ponds/reservoirs, building/using walkways, reinforcing or constructing strong buildings, use of sand bags for flood breaks, water pumping machines, demi-lunes, draining water bodies, canoes, expansion/construction of sanitation network, gabions, permeable pavements, reinforcing infrastructure, construction of basic infrastructure, hill slopes, pumps, stone bonding, ridges across slopes, digging of boreholes, using generators, mud heaps, building bridges, watertight trenches, breakwater systems, closure of dam, land reclamation
	Mutual support	Support from community/social environment, social relations, formation of associations and groups, advocating for disadvantaged groups, volunteer groups
	Maintenance activities	Clearing drainage, waste management, maintaining existing flood drainage infrastructure, clean-up activity, reconstruction and rehabilitation, repair activity, removing water out of flooded area, better waste management, procuring lost items, better waste management behaviour, improved sanitation, ensuring continuation of household activity, update flood control measures, maintenance of existing flood defense systems, recover lost livestock
	Awareness-raising, training and education	Civic sensitisation to flood risk, warning campaigns in media, raising awareness to improve waste management behaviour, capacity-building of staff, provision of alternative skill development, women empowerment programme, teaching of coping and adaptation skills, public health education, enhancing education, raising awareness on the need of obtaining building permits, increase volunteer participation, health inspectors
	Information resources	Early warning systems, weather information/forecasts, establishment of a Geographic Information System (GIS), looking for flood information on the news/social media, radio/TV/phone ownership, collaborate for media coverage of the event, reliance on extension information, better warning/risk communication, forecasting, accessibility of weather and environmental information, seeking access to information sources, credible sources of information
	(Preparing/providing) assistance and response	Raising response capacities/relief activities, governmental assistance, assistance from NGOs/relief organisations, establishment of emergency/contingency plans, risk management committees, storing food, coordination of disaster responses, formal loans, drills, preparing for power cuts, keeping medical kit in the household, provision of relief items, provision of shelters, assistance from community-based organisations, assistance from religious institutions, assistance from private companies, creation of employment, emergency preparedness mechanisms, coordination of flood response, acquiring pumps for houses, personal preparations, credit access, encouraging risk management at the village/community level, emergency drills, creation of an emergency response agency, transportation in case of emergency, preparing for power cuts, creating safe zones, extend governmental response from urban to rural areas
	Relocation	Permanent relocation, temporary relocation, forced eviction and resettlement from flood areas, moving items/animals to a safe place, farming in higher areas, avoid farming in exposed zones, migration
	Spatial planning interventions	Formalisation of informal settlements, restructuration of areas, creation of social housing, participative planning, flood-related land use planning, enforcement of land use laws/demolition, monitoring implementation of flood-reducing infrastructure, more integrated land use planning, urban upgrading programmes, incentives for people to move out of flood zones, environmental management, better building codes, provision of sanitation, investing in other areas apart from the capital
	Use of local knowledge and skills	Local knowledge of floods, sharing technical knowledge, employing more qualified staff, staying alert, appreciation of local/traditional knowledge in disaster risk management (DRM), organisation and leadership
	Policies	Better integration of groups at risk, active collaboration among stakeholders, policies which alter the resources of people at risk, assignment of clear responsibilities, law and policy enforcement, adjusting policies to local context, formalisation of exchange between actors, decentralisation of agencies/DRR capacities, more funds for DRR, environmental management policies, public policies to reduce flood risk, creation of development/response agencies, provision of funds for DRR, tolerance/formalisation of informal settlements, decentralisation of agencies, sanitation laws, transfer of responsibilities to lower level government bodies, institutional reforms, cooperation with private entities, enhancing institutional capacity, international cooperation, long-term orientation of policies, audit on corruption prevention
	Insurance	Obtaining insurance cover, receiving compensation from insurance
	Nature-based solutions	Wetland conservation, afforestation, mangroves, agroforestry, urban greening, use of flood plains to retain water, greening of lands, consume wild fruits and plants, protecting and using natural barriers, burning of fruit peels to drive away mosquitoes, rehabilitating/protecting wetlands, natural reserves in high-risk areas, green and hybrid measures, reducing environmental degradation
	Research and assessment	Research on potential risk-reducing measures, consider social aspects of flood risk, mapping of flood zones, hydrological data collection, risk assessments/mapping, hazard modelling, institutional assessments, flood risk research, humanitarian/situation assessment, research on causal interaction in disaster risk, establishing research cooperations, participatory research, data collection on impact measurements, collect population data, more research, monitoring urban expansion
	Modification of practices	Modified agricultural techniques, change of water supply practices, switching off gas and electricity, avoiding movement, consuming less meals, using rain boots, supervising children, dependence on market for food, conflict resolution, hire security guard, modified washing behaviour, trying to retrieve the rent, living in one room only, water harvesting, practice intense fishing system, sharing of family responsibilities between women and men, switching off gas and electricity, product pooling of produce
	Risk retention/using retained resources	Staying in flooded house/area, saving/use of savings, inactivity, consume stored food, emergency funds
	Modification of livelihood	Non-agricultural activities, diversification of economic activities, fishing, market gardening, additional employment, buy livestock, selling goods/assets, mutual exchanges/trade, creation of income generating activities, renting out exposed house, encourage artisanal jobs, encourage seed exchanges, selling/renting new land titles
	Religious and spiritual activities	Religious beliefs, prayers/fasting, spiritual support, religious support with social safety nets
	Health care	Provision of (affordable) health care, self-medication, use of insect sprays/mosquito nets, medication, application of traditional medicine, develop better health centres, sanitising flood water, visiting midwives, sanitation following hygiene rules, water treatment, psychological support
Impacts from residual flood risks from the most recent flood event	Material damage	Damaged/destroyed buildings, damaged possessions/goods, damage to infrastructure, crop damage, loss of livestock, damage of public facilities, destruction of processed goods/produce, damage to farms, reduction of fish catch
	Health	Fatalities, sickness and spread of diseases, fear/mental health problems, injuries, general status of poor health, malnutrition, no immediate health care, miscarriages
	Economic losses	Disruption of livelihoods/income loss, financial damages, poverty and uncertainty
	Environmental degradation	Damaged farming land/land degradation, polluted environment, loss/disturbance of ecosystems
	Displacement and homelessness	Displacement, homelessness
	Lack of food/drinking water	Lack of drinking water/water contamination, lack of food/scarcity
	Lack of mobility	Disruption of general movement, traffic interruption
	Interruption of social activities	Interruption of education, negative impacts on social life, crime/theft/violence/conflicts

## Results

### Meta-information

**Fig. 3:**
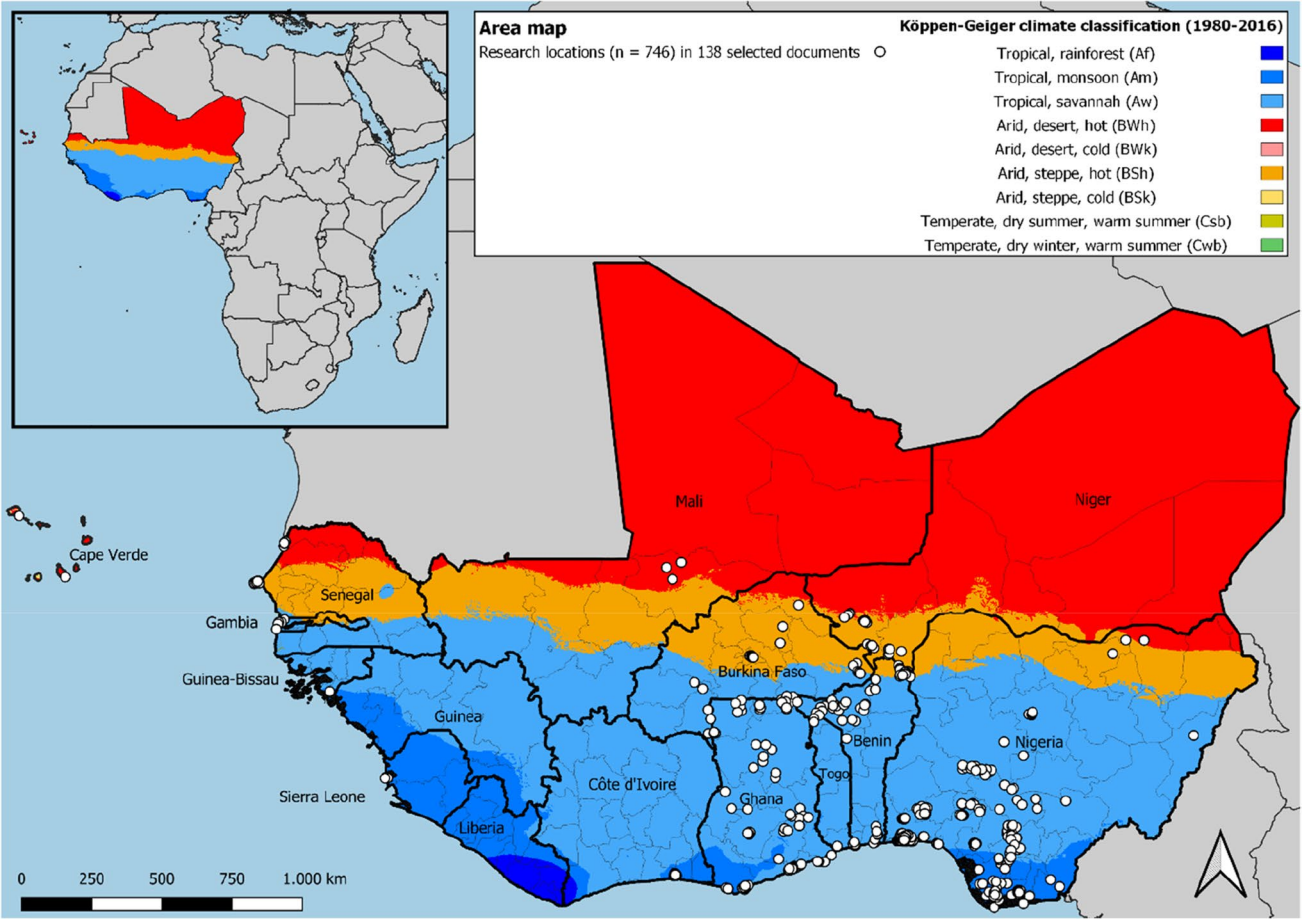
Geographical distribution of research locations in selected documents. The authors excluded publications [73] and [81] (see Annex 3) from the map due to not specifying the research locations sufficiently. One article can contain several research areas, resulting in 746 research locations from 138 selected documents. Admin boundaries retrieved from DIVA GIS (2020) and Köppen-Geiger climate classification data set from Beck et al. (2018)

The review analysis showed that the number of FRM-related articles has steadily increased from 2011 onward (Annex 3) and that the majority of selected articles mentioned Nigeria and Ghana as research areas (Annex 4). Those countries are followed by Senegal, Benin, Niger, Burkina Faso, Togo, and Ivory Coast. Furthermore Cape Verde, The Gambia, Sierra Leone, Mali, and Guinea-Bissau are countries that only featured once or twice in the selected articles. It is worth noting that the final set of selected articles did not represent Guinea and Liberia. Since most articles focussed on Nigeria and Ghana and urban or peri-urban areas (Annex 5), a bias towards those geographical areas must be considered in the results obtained. Furthermore, the geographic distribution of research areas was mapped (see Fig. 3). The map illustrates that, according to the Köppen-Geiger classification from 1980–2016 (Beck et al. 2018), the research area spans nine different climatic zones, of which the following five cover the majority of this area: tropical, rainforest (Af); tropical, monsoon (Am); tropical, savannah (Aw); arid, desert, hot (BWh); and arid, steppe, hot (BSh). It became apparent that the eastern part of the region is widely covered by the selected research. In contrast, the western part is barely covered, with the exception of the Senegalese coast, The Gambia and singular studies in Cape Verde, Guinea-Bissau, and Sierra Leone. Flood types that were encountered in the review (Annex 6) were pluvial floods *(n* = *93)*, fluvial floods *(n* = *83)*, coastal floods *(n* = *34)*, and groundwater floods *(n* = *9)*. The variety of methods applied in case studies also translates into a varying understanding of concepts that relate to FRM such as risk, vulnerability, adaptation, or coping. Methods for primary data collection (Annex 7) were surveys *(n* = *97)*, qualitative interviews (semi-structured, in-depth, and key-informant) *(n* = *73)*, focus groups *(n* = *40)*, photography/photo-elicitation *(n* = *13)*, workshops *(n* = *11)*, stakeholder meetings *(n* = *10)*, transect walks *(n* = *6)*, and collective mapping *(n* = *4)*. The following part of the section will summarise the information collected in the review process, based on the four research questions previously stated in chapter 1.

### Which existing risk-reducing measures were mentioned before the onset of the most recent flood event?

**Fig. 4:**
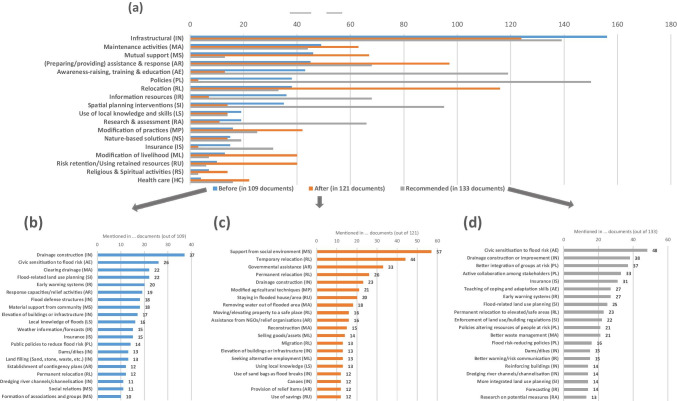
Overview of categories (a) and measures before (b) and after (c) the onset of the most recent flood event as well as (d) recommended measures by FRM-related research (one document can contain several categories and measures)

The analysis shows that observed FRM measures that were mentioned before the most recent flood event (appearing in 109 out of 138 documents) most often fell into the category of infrastructural measures (Fig. 4a), with drainage construction being the most outstanding among them (Fig. 4b). Also, flood defense structures, elevating of buildings or infrastructure, landfilling, dams/dikes, and dredging of rivers/channelisation were mentioned as infrastructural measures. Following infrastructural measures, a cluster of six categories of risk management measures before the onset of the most recent flood event showed an equal prevalence. This comprises the following categories (Fig. 4a) and measures (Fig. 4b): maintenance activities with measures such as clearing drainage infrastructure; mutual support with measures such as material support from the community and social relations; preparing/providing assistance and response with measures such as raising capacities for response and relief and the establishment of contingency plans; awareness-raising, training, and education with measures such as civic sensitisation to flood risk; policies with measures such as applying public policies to reduce flood risk and a flood control/development master plan; and, finally, relocation with measures such as permanent relocation.

### Which impacts from the most recent flood event were mentioned in selected case studies?

**Fig. 5:**
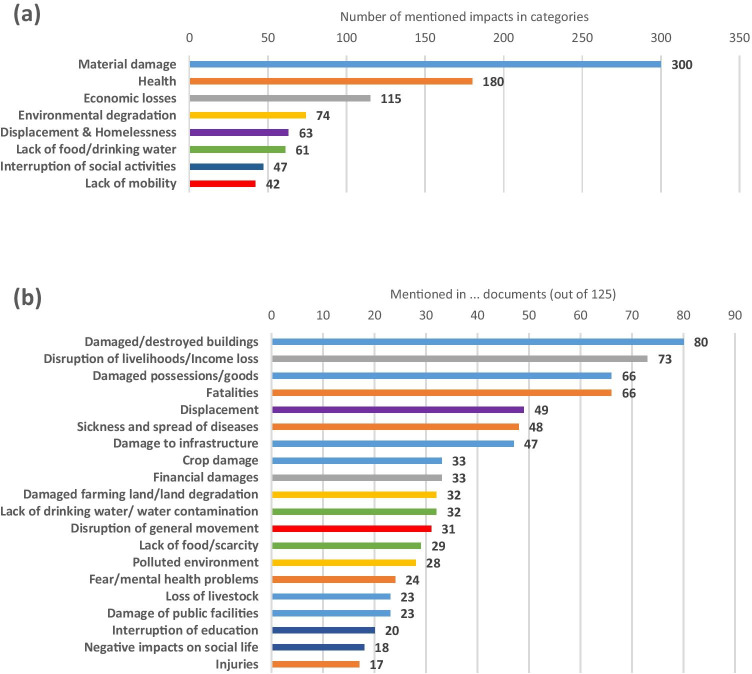
Categories of mentioned flood impacts from residual flood risks (a) and flood impacts (b) (one document can contain several categories and impacts)

Impacts from residual flood risk were analysed, which arose from the most recent flood event despite FRM measures or in their absence (appearing in 125 out of 138 documents). The results demonstrate that in the selected documents, flood impacts most frequently fall into the category of material damage (Fig. 5a) due to, for example, damaged buildings as the most outstanding impact, damaged possessions, damage to infrastructure, crop damage, loss of livestock, and damage of public facilities (Fig. 5b). However, health impacts *(n* = *180)* also pose a significant risk resulting from a flood event in analysed case studies. They mostly materialise as fatalities, sickness, and spreading of disease, as well as fear/mental health problems. Besides, economic losses *(n* = *115)* are frequent impacts resulting from flood events in analysed case studies. They often took the form of disruption of livelihoods/income loss, and financial damages. Additionally, environmental degradation *(n* = *74)* played an important role in impacts which resulted from the most recent flood event in the selected documents. These impacts often resulted in damaged farming land/land degradation and a polluted environment. Finally, displacement and homelessness, lack of food/drinking water, interruption of social activities, and lack of mobility emerged as dimensions of flood impacts worth considering.

### Which measures were applied to deal with impacts from residual flood risk after the onset of the most recent flood event?

The following paragraph summarises measures that were applied to deal with impacts from residual flood risk after the onset of the most recent flood event (appearing in 121 out of 138 documents). Similarly to before the onset of the most recent flood event, infrastructural measures were performed most frequently after its onset (Fig. 4a). They often appeared as belated drainage/channel construction or by using sandbags as flood breaks (Fig. 4c). Also, measures of relocation *(n* = *116)* were performed very frequently after the most recent flood event had started. In comparison to before the onset of the flood event, they strongly increased after its onset. These measures unfolded as temporary relocation, permanent relocation, moving/elevating property to a safe place, and migration. Also, measures of mutual support played a highly important role after the onset of the most recent flood event *(n* = *67)*. These measures were reported for example as receiving support from the social environment. It is worth noting that this measure was the most frequent after the onset of the most recent flood event. Despite being stated vaguely in many publications, some specified such mutual support activities as providing labour, mental, financial, or material support, borrowing money, and food or shelter to affected family members or friends. Moreover, reported measures focussing on providing/preparing assistance and a response played a crucial role after the onset of the flood event *(n* = *97)*. They were performed as governmental assistance, assistance from NGOs/relief organisations, or in general as provision of relief items. Compensations received from insurance companies did not play a significant role.

### Which FRM measures were recommended to address residual flood risk?

Finally, measures that were recommended in selected documents to address residual flood risk were identified in 133 out of 138 documents. In contrast to practiced measures before and after the onset of the flood event, measures to adjust policies *(n* = *150)* were most frequently recommended by selected documents to deal with residual flood risk (Fig. 4a). Such adjustments were recommended to better integrate groups at risk into decision-making, active collaboration among stakeholders, policies that alter the resources of people at risk, and policies which directly reduce flood risk (Fig. 4d). Aside from being widely practiced before and after the onset of the most recent flood event, infrastructural measures were again highly recommended *(n* = *139)* for further risk reduction efforts. Other recommended measures comprise, for example, of drainage construction or improvement, dams/dikes, reinforcing buildings, and dredging river channels/channelisation. Additionally, more effort towards measures aimed at awareness-raising, training, and education *(n* = *119)* were recommended by many selected documents. For example, those comprised of further efforts for civic sensitisation to flood risk and teaching of skills to cope with and adapt to floods. Interestingly, despite the fact that not many documents focussed on insurance explicitly in their assessments, it appears as the fifth-most frequently recommended measure.

## Discussion

The academic literature analysed in this paper pinpoints the dimensions of impacts that resulted from residual flood risk for the West African region. They comprised most prominently material damage, health impacts, and economic losses, but also environmental degradation, displacement and homelessness, lack of food/drinking water, interruption of social activities, and lack of mobility. It is worth noting that the term “residual risk” was mentioned only once (Adelekan 2016) and not subject to direct analysis in any of the selected documents. Thus, the concept of residual risk has not yet been taken up in selected FRM-related literature. Material damage appeared to be a dominant category of impacts from residual flood risk (Fig. 5a) in selected research according to the analytical approach of this review (Fig. 1). Considering other types of impacts from residual flood risk identified by this review article more extensively will enrich the perspective of FRM. The most prevalent additional dimensions were health impacts and economic losses, which are also currently receiving increased attention due to being the most discussed impacts in the ongoing COVID-19 pandemic (Nicola et al. 2020; Holmes et al. 2020; El Zowalaty and Järhult 2020). This observation gains additional relevance regarding the low level of health care efforts to address flood impacts (Fig. 4a) as well as the high activity to modify livelihoods after the onset of the most recent flood event identified in this review (e.g. Ajibade et al. 2013, Hetcheli 2013, Schaer 2015, Ajaero 2017, Oyerinde et al. 2017, Markantonis et al. 2018, Atidegla et al. 2018, Afriyie et al. 2018; and Fig. 4a). The political momentum in ECOWAS countries for addressing the health and economic impacts of the COVID-19 pandemic (IMF 2020) could help to pursue the possibility of joining efforts in reducing the risk of impacts from both floods and pandemics. In doing so, the consideration of fear and mental health problems arising from either traumatic flood experiences or pandemics should not be neglected. Also, the various dimensions of flood impacts resulting from residual risk underscore the need for research that assesses the causal chains of flood impacts and their mutual influence on each other.

Moreover, the review elaborated that infrastructural measures have been the most observed category of measures in the selected case studies (Fig. 4a). The emphasis on infrastructural measures in FRM-related research is further underscored by a rare explicit application of nature-based solutions, as well as of recommendations for it (Fig. 4a). The tendency for implementing infrastructural measures could be observed before (e.g. Campion and Venzke 2013; Odemerho 2015; Adelekan 2016; Kablan et al. 2019) and after (e.g. Mbow et al. 2008, Schaer and Hanonou 2017, Owusu Twum and Abubakari 2019, Bottazzi et al. 2019) the most recent flood event. Still, infrastructural measures were often recommended in FRM-related research (e.g. Saidu 2009, Adewole et al. 2015, Serpantié et al. 2019; and Fig. 4a). The latter finding could point towards the inadequacies of existing systems, such as open drainage facilities blocked by waste (e.g. Lokonon 2016; Osayomi and Oladosu 2016; Danso and Addo 2017; Schlef et al. 2018) or having to resort to isolated efforts of flood defense structures on the house- or community-level, often in informal areas, with limited impact (Adelekan 2010; Schaer 2015; Bottazzi et al. 2018; Adekola et al. 2019). This was frequently mentioned in urban case studies. Also, the prevalence of recommendations for spatial planning interventions (Fig. 4a) has to be understood in light of the limitations of infrastructural measures. Frequently mentioned measures were, for example, improved land use planning which better considers flood risk (e.g. Wahab and Falola 2017; Tiepolo et al. 2019) or the enforcement of existing land use plans to avoid the new construction of buildings of infrastructure in high-risk areas (e.g. Onu et al. 2013; Ibitoye et al. 2019). However, it seems to remain a difficult task, regarding projections for urban expansion along the Niger river and low-elevation coastal zones (LECZ) along the Gulf of Guinea up to 2030 (Güneralp et al. 2015).

Regarding the polycentral and participatory approach of FRM, there appears to be a strong need for more participatory and inclusive governance to further reduce the impacts of residual flood risk further, given the strong recommendation by the selected documents for policy and law-related measures (Fig. 4a). Those recommendations are often pointed towards better collaboration among stakeholders (e.g. Olokesusi et al. 2015; Ntajal et al. 2017; Young et al. 2019), better integrating groups at risk in relation to decision-making (e.g. Komi et al. 2016; Frick-Trzebitzky and Bruns 2019), and altering their resources (e.g. Olanrewaju et al. 2019, Cirella et al. 2019). This need is also reflected in the current relative disregard of local knowledge and skills in dealing with floods (Fig. 4a). Hence, future research projects should include a focus on how widely present and existing local knowledge and skills could be better integrated into decision-making processes in a meaningful way (e.g. Bonye and Godfred 2011; Biconne 2014; Ajibade and McBean 2014; Ngwese et al. 2018). It has also become apparent in this review that the documents identified civic sensitisation to flood risk as a priority action area for further efforts in flood risk reduction (e.g. Agbola et al. 2012, Adeleye and Ayangbile 2016, Ottah 2017, Abass et al. 2019; and Fig. 4d). Such measures may include early warning systems, as they also appeared as a frequently recommended measure (e.g. Coker et al. 2014, Vissin et al. 2016, Egbinola et al. 2017; and Fig. 4d). The need for this could be further enlarged by expected climatic changes for West Africa, which are projected to lead to shorter yet more intense rainy seasons (Vizy and Cook 2012; Dunning and Black 2018; Akinsanola and Zhou 2019; Dosio et al. 2019).

Remarkably, the most widely practiced measure after the onset of a recent flood event was to seek support from the social environment (Fig. 4c). Whilst some documents did not define the measures more precisely (e.g. Boamah et al. 2015; Enete et al. 2016; Evadzi et al. 2018), others explicitly indicated them as providing labour, mental, financial, or material support, borrowing money, and food or shelter to affected family members or friends (e.g. Adelekan and Fregene 2015; Kielland 2016; Osman et al. 2016; Frick-Trzebitzky 2017; Ajaero et al. 2018). Thus, support from social networks can also aid in explaining the strong prevalence of temporary relocation after the onset of the flood events in case studies (Fig. 4c). Whilst indicating a high level of solidarity, the strong support within social networks also illustrates a lack of widespread access to or compensation by insurance schemes. More research could look into the types of risks shared in such social networks, their limitations, and which form of support aids in the recovery process. It is also worth exploring how efficient and effective those networks function in addressing residual flood risk, if the networks help alleviate inequality and if they are fair on their members. It could be of further interest if those social networks even take on the form of informal risk transfer arrangements, in which support is provided in exchange for social or financial benefits (UNDRR 2020b). This aspect is particularly interesting since many documents recommended insurance for further residual flood risk reduction, despite only a few providing an explicit assessment of its suitability or usage (e.g. Oyekale et al. 2013; Antwi-Boasiako 2016, 2017; Osayomi and Oladosu 2016; Glago 2019). Thus, exploring if insurance can be helpful to address residual flood risk whilst considering the presence of existing informal arrangements appears highly relevant in this research context.

## Conclusion

As floods in the West African region have become increasingly frequent and devastating in the past decades, it is essential to give an account of which FRM measures and impacts from residual flood risk are primarily addressed in academic literature. This review found residual risk and its management to be treated implicitly, if at all. An explicit focus is missing in the current FRM-related research carried out in West Africa and will deserve more attention in future. Also, the review identifies that FRM measures frequently comprise of infrastructural measures, maintenance activities, mutual support (in particular seeking support from the social environment), and the preparation/provision of assistance and response measures both before and after the most recent flood event mentioned in case studies. Among those, infrastructural measures emerged as dominant FRM component in this review. Besides, temporary and permanent relocation activities were frequently observed after the onset of the most recent flood event in selected documents. In addition, recommendations provided in selected documents to reduce residual flood risk were mainly categorised as adjustments of policies, infrastructural measures, awareness-raising, training and education, and spatial planning interventions.

Furthermore, certain limitations of the study should be observed. It was beyond the scope of the review to collect information on the effectiveness and efficiency of individual measures. Besides, additional relevant research might be published in other databases beyond those considered for this review (Web of Knowledge, Scopus, and African Journals Online). In addition, most analysed research was carried out in only a few countries (Nigeria and Ghana) and specific geographical areas (urban + peri-urban and coastal). This aspect affects the generalisability of the results for the entire West African region. Consequently, future research should consider other potentially flood-affected countries and areas that have as well remained neglected by existing research so far. Therefore, analyses could assess if the spatial distribution of FRM-related research reflects the spatial distribution of flood impacts in ECOWAS countries, by for example drawing upon data from the EM-Dat database. Finally, the varying understanding of concepts relating to FRM such as risk, vulnerability, adaptation, or coping has to be observed when summarising such information on a meta-level. However, it is beyond the scope of this review to compare and contrast those variations.

Future studies could either embark on more complex modelling that approaches residual flood risk by researching the synergies of FRM-measures, their limitations in reducing the risk of flooding, and the various dimensions of impacts that arise from it. Or, as applied in this review, a focus on flood impacts that occur despite the implementation of FRM measures could also enrich case studies to approach residual flood risk from an empirical perspective. Moreover, more research on the role of social networks in the recovery from flood impacts, the range of impacts they usually cover, and the conditions that prevail within them will be highly relevant. It will also be necessary to research if and to what extent financial damages are covered and if those arrangements qualify as risk transfer mechanisms. Such research will help devise locally appropriate mechanisms that help address flood impacts that put people in financial need. Those efforts should be coupled with more thorough and detailed assessments of the suitability of insurance in addressing residual flood risk, given its currently limited role. Besides, future research could acknowledge the strong prevalence of infrastructural measures by investigating the problems that appear in implementing adequate flood-reducing infrastructure more deeply and how to overcome them. In addition, it could be relevant to research to what extent such measures could be complemented or substituted by nature-based solutions, which currently do not play a role in FRM-related West African case studies yet. Furthermore, the body of selected literature strongly raised the need for more participatory approaches that ensure the involvement of the population at-risk in decision-making and research. Such efforts could be focussed on but not limited to spatial planning interventions, awareness-raising training and education, and infrastructure construction. Finally, the use of local knowledge and skills in the form of FRM measures that the at-risk population already practices portrays another opportunity for such involvements. However, the latter aspect is not part of the dominant foci of practiced or recommended measures that this review identified but still should be subject to future research.

## Supplementary Information

Below is the link to the electronic supplementary material.

Supplementary file1 (DOCX 316 KB)
