# Supplementary material for: When does risk become residual? A systematic review of research on flood risk management in West Africa
Source: Reg Environ Change. 2021 Aug 25;21(3):84. doi: 10.1007/s10113-021-01826-7 (PMC8384556; doi:10.1007/s10113-021-01826-7)
Supplement: Supplementary file 1 — Supplementary file1 (DOCX 316 KB) [file 10113_2021_1826_MOESM1_ESM.docx]

***When does risk become residual? - A systematic review of flood risk management-related academic literature in West Africa***

***Simon Wagner^1 2^, Maxime Souvignet^1^, Yvonne Walz^1^, Kehinde Balogun^1^, Kossi Komi^3^, Sönke Kreft^1^, Jakob Rhyner^2^***

*^1^ United Nations University – Institute for Environment and Human Security (UNU-EHS), UN Campus Platz der Vereinten Nationen 1, D-53113 Bonn, Germany*

*^2^ Agricultural Faculty, University of Bonn, Meckenheimer Allee 174, 53115 Bonn, Germany*

*^3^ Laboratoire de Recherche sur les Espaces, les Echanges et la Sécurité Humaine, Département de Géographie, Université de Lomé, BP: 1515, Lomé, Togo*

[*s.wagner@ehs.unu.edu*](mailto:s.wagner@ehs.unu.edu)*;* [*souvignet@ehs.unu.edu*](mailto:souvignet@ehs.unu.edu)*;* [*walz@ehs.unu.edu*](mailto:walz@ehs.unu.edu)*;* [*balogun@ehs.unu.edu*](mailto:balogun@ehs.unu.edu)*;* [*kossik81@yahoo.fr*](mailto:kossik81@yahoo.fr)*;* [*kreft@ehs.unu.edu*](mailto:kreft@ehs.unu.edu)*;* [*rhyner@uni-bonn.de*](mailto:rhyner@uni-bonn.de)

Submission of article to Regional Environmental Change

**Supplementary material**

**Annex 1** Search terms used in Web of Knowledge, Scopus, and African Journals Online (AJOL)

**Web of Knowledge:** (TS=(flood* OR *inond* OR inundat* OR crue*) AND TS=(*risk* OR risque* OR residual OR résiduel* OR management OR gestion OR vulnerab* OR vulnérab* OR adapt* OR resilien* OR résilien* OR cop* OR "faire face" OR mitigat* OR atténu* OR reduc* OR réduc* OR impact* OR loss* OR perte$ OR damage$ OR dégâts OR respon* OR répon* OR disast* OR catastroph* OR capacit* OR protect* OR warn* OR alert* OR transfer OR transfert OR retention OR rétention OR insur* OR assur* OR reinsur* OR réassur* OR remittance* OR versement* OR aid$ OR help* OR mutu?l* OR gift* OR cadeau* OR shar* or partag* OR cr?dit* OR fund* OR fonds OR r?serve$ OR saving* OR économi* OR income$ OR revenu$ OR poverty OR poor* OR pauvre* OR livelihood$ OR subsistence OR agricultur* OR bank$ OR banque$ OR government* OR gouvernement* OR famil* OR commun* OR network$ OR réseau$) AND TS=("west* africa*" OR "afrique de l'ouest" OR "ouest-africain*" OR benin* OR bénin* OR togo* OR senegal* OR sénégal* OR gambia* OR gambie* OR guinea* OR guinée* OR "guinea-bissau*" OR "guinée-bissau*" OR "bissau-guinéen*" OR mali* OR "ivory coast" OR ivorian* OR "côte d'ivoire" OR ivoirien* OR "sierra leone*" OR "sierra léon*" OR "burkina faso" OR burkinab* OR niger* OR nigér* OR nigeria* OR nigéria* OR ghan* OR liberia* OR libérien* OR "cap-ver*" OR "cape verd*") NOT TS=(malicious OR malignant OR "Papua New Guinea" OR attack*)) AND LANGUAGE: (English OR French) AND DOCUMENT TYPES: (Article OR Book Chapter) Indexes=SCI-EXPANDED, SSCI, A&HCI, ESCI Timespan=1991-2019-> **1,222 documents**

**Scopus:** TITLE-ABS-KEY ( flood* OR *inond* OR inundat* OR crue* ) AND TITLE-ABS-KEY ( *risk* OR risque* OR residual OR résiduel* OR management OR gestion OR vulnerab* OR vulnérab* OR adapt* OR resilien* OR résilien* OR cop* OR "faire face" OR mitigat* OR atténu* OR reduc* OR réduc* OR impact* OR loss* OR perte$ OR damage$ OR dégâts OR respon* OR répon* OR disast* OR catastroph* OR capacit* OR protect* OR warn* OR alert* OR transfer OR transfert OR retention OR rétention OR insur* OR assur* OR reinsur* OR réassur* OR remittance* OR versement* OR aid$ OR help* OR mutu?l* OR gift* OR cadeau* OR shar* OR partag* OR cr?dit* OR fund* OR fonds OR r?serve$ OR saving* OR économi* OR income$ OR revenu$ OR poverty OR poor* OR pauvre* OR livelihood$ OR subsistence OR agricultur* OR bank$ OR banque$ OR government* OR gouvernement* OR famil* OR commun* OR network$ OR réseau$ ) AND TITLE-ABS-KEY ( "west* africa*" OR "afrique de l'ouest" OR "ouest-africain*" OR benin* OR bénin* OR togo* OR senegal* OR sénégal* OR gambia* OR gambie* OR guinea* OR guinée* OR "guinea-bissau*" OR "guinée-bissau*" OR "bissau-guinéen*" OR mali* OR "ivory coast" OR ivorian* OR "côte d'ivoire" OR ivoirien* OR "sierra leone*" OR "sierra léon*" OR "burkina faso" OR burkinab* OR niger* OR nigér* OR nigeria* OR nigéria* OR ghan* OR liberia* OR libérien* OR "cap-ver*" OR "cape verd*" ) AND PUBYEAR > 1990 AND PUBYEAR < 2020 AND LANGUAGE ( english OR french ) AND DOCTYPE ( ar OR ch ) AND NOT TITLE-ABS-KEY ( malicious OR malignant OR "Papua New Guinea" OR attack* ) -> **1,539 documents**

**African Journals Online (AJOL) (earliest date possible in this database is 2004):**

(flood* OR *in?nd* OR crue*) AND (*risk* OR risque* OR r?sidu?l* OR management OR gestion OR vuln?rab* OR adapt* OR r?silien* OR cop* OR "faire face" OR mitigat* OR atténu* OR r?duc* OR impact* OR loss* OR perte$ OR damage$ OR dégâts OR r?$pon* OR disast* OR catastroph* OR capacit* OR protect* OR warn* OR alert* OR transfer$ OR r?tention OR insur* OR assur* OR reinsur* OR réassur* OR remittance* OR versement* OR aid$ OR help* OR mutu?l* OR gift* OR cadeau* OR shar* OR partag* OR cr?dit* OR fund* OR fonds OR r?serve$ OR saving* OR économi* OR income$ OR revenu$ OR poverty OR poor* OR pauvre* OR livelihood$ OR subsistence OR agricultur* OR bank$ OR banque$ OR go$vern$ment* OR famil* OR commun* OR network$ OR réseau$)

Search period: 01/01/2004 – 31/12/2019 -> **173 documents**

__________________________________________________________________________________

**Annex 2** List of selected documents

**[1] Abass, K.; Dumedah, G.; Frempong, F. (2019):** Understanding the physical and human contexts of fluvial floods in rural Ghana. In International Journal of River Basin Management, pp. 1–12. DOI: 10.1080/15715124.2019.1653310.

**[2] Abu, M.; Codjoe, S. (2018):** Experience and future perceived risk of floods and diarrheal disease in urban poor communities in Accra, Ghana. In International Journal of Environmental Research and Public Health 15, p. 2830. DOI: 10.3390/ijerph15122830.

**[3] Addo, I. Y.; Danso, S. Y. (2017):** Sociocultural factors and perceptions associated with voluntary and permanent relocation of flood victims: A case study of Sekondi-Takoradi Metropolis in Ghana. In Jàmbá: Journal of Disaster Risk Studies 9 (1), p. 303. DOI: 10.4102/jamba.v9i1.303.

**[4] Adebo, G.; Ayelari, T. (2011):** Climate change and vulnerability of fish farmers in Southwestern Nigeria. In African Journal of Agricultural Research 6.

**[5] Adejuwon, G. A.; Aina, W. J. (2014):** Emergency preparedness and response to Ibadan flood disaster 2011: Implications for wellbeing. In Mediterranean Journal of Social Sciences, pp. 500–511. DOI: 10.5901/mjss.2014.v5n8p500.

**[6] Adekola, O.; Lamond, J.; Adelekan, I.; Eze, E. B. (2019):** Evaluating flood adaptation governance in the city of Calabar, Nigeria. In Climate and Development, pp. 1–14. DOI: 10.1080/17565529.2019.1700771.

**[7] Adelekan, I. O. (2010):** Vulnerability of poor urban coastal communities to flooding in Lagos, Nigeria. In Environment and Urbanization 22 (2), pp. 433–450. DOI: 10.1177/0956247810380141.

**[8] Adelekan, I. O. (2011):** Vulnerability assessment of an urban flood in Nigeria: Abeokuta flood 2007. In Natural Hazards 56 (1), pp. 215–231. DOI: 10.1007/s11069-010-9564-z.

**[9] Adelekan, I. O. (2016):** Flood risk management in the coastal city of Lagos, Nigeria. In Journal of Flood Risk Management 9 (3), pp. 255–264. DOI: 10.1111/jfr3.12179.

**[10] Adelekan, I. O.; Asiyanbi, A. P. (2016):** Flood risk perception in flood-affected communities in Lagos, Nigeria. In Natural Hazards 80 (1), pp. 445–469. DOI: 10.1007/s11069-015-1977-2.

**[11] Adelekan, I. O.; Fregene, T. (2015):** Vulnerability of artisanal fishing communities to flood risks in coastal southwest Nigeria. In Climate and Development 7 (4), pp. 322–338. DOI: 10.1080/17565529.2014.951011.

**[12] Adeleye, B.; Popoola, A. (2019):** Poor development control as flood vulnerability factor in Suleja, Nigeria. In Town and Regional Planning 74 (1), pp. 23–35. DOI: 10.18820/2415-0495/trp74i1.3.

**[13] Adeleye, B. M.; Ayangbile, O. A. (2016):** Flood vulnerability: Impending danger in Sabon-Gari Minna, Niger State, Nigeria. In Ethiopian Journal of Environmental Studies and Management 9 (1), pp. 35–44. DOI: 10.4314/ejesm.v9i1.4.

**[14] Adewole, I. F.; Agbola, S. B.; Kasim, O. F. (2015):** Building resilience to climate change impacts after the 2011 flood disaster at the University of Ibadan, Nigeria. In Environment and Urbanization 27 (1), pp. 199–216. DOI: 10.1177/0956247814547679.

**[15] Afriyie, K.; Ganle, J. K.; Santos, E. (2018):** ‘The floods came and we lost everything’: weather extremes and households’ asset vulnerability and adaptation in rural Ghana. In Climate and Development 10 (3), pp. 259–274. DOI: 10.1080/17565529.2017.1291403.

**[16] Agbola, B. S.; Ajayi, O.; Taiwo, O. J.; Wahab, B. W. (2012):** The August 2011 flood in Ibadan, Nigeria: Anthropogenic causes and consequences. In International Journal of Disaster Risk Science 3 (4), pp. 207–217. DOI: 10.1007/s13753-012-0021-3.

**[17] Ahadzie, D. K.; Dinye, I.; Dinye, R. D.; Proverbs, D. G. (2016):** Flood risk perception, coping and management in two vulnerable communities in Kumasi, Ghana. In International Journal of Safety and Security Engineering 6 (3), pp. 538–549. DOI: 10.2495/SAFE-V6-N3-538-549.

**[18] Ahmed, S. D.; Agodzo, S. K.; Adjei, K. A.; Deinmodei, M.; Ameso, V. C. (2018):** Preliminary investigation of flooding problems and the occurrence of kidney disease around Hadejia-Nguru wetlands, Nigeria and the need for an ecohydrology solution. In Ecohydrology & Hydrobiology 18 (2), pp. 212–224. DOI: 10.1016/j.ecohyd.2017.11.005.

**[19] Ajaero, C. K. (2017):** A gender perspective on the impact of flood on the food security of households in rural communities of Anambra state, Nigeria. In Food Security 9 (4), pp. 685–695. DOI: 10.1007/s12571-017-0695-x.

**[20] Ajaero, C. K.; Mozie, A.; Abu, I. (2018):** Migrating from migratory waters to migration of livelihoods. In Social Indicators Research 136, pp. 1–15. DOI: 10.1007/s11205-016-1524-x.

**[21] Ajaero, I. D.; Okoro, N. M.; Ajaero, C. K. (2016):** Perception of and attitude toward mass media reportage of the 2012 flood in rural Nigeria. In SAGE Open 6 (3), 215824401666688. DOI: 10.1177/2158244016666887.

**[22] Ajaero C.K., Mozie A.T., Anaelo C.N. (2018):** Gender mainstreaming of the impacts of 2012 flood-induced migration on household livelihoods in Nigeria. In International Journal of Sustainable Development 21 (1/2/3/4), pp. 18–35.

**[23] Ajibade, E. T.; Babatunde, R. O.; Ajibade, T. B.; Akinsola, G. O. (2019):** Empirical analysis of adaptation strategies used in mitigating flood related losses by rice farmers in Kwara State, Nigeria. In Agrosearch 19 (1), pp. 59–71. DOI: 10.4314/agrosh.v19i1.5.

**[24] Ajibade, I.; Armah, F.; Kuuire, V.; Luginaah, I.; McBean, G. (2015a):** Self-reported experiences of climate change in Nigeria: The role of personal and socio-environmental factors. In Climate 3 (1), pp. 16–41. DOI: 10.3390/cli3010016.

**[25] Ajibade, I.; Armah, F.; Kuuire, V.; Luginaah, I.; McBean, G.; Tenkorang, E. (2015b):** Assessing the bio-psychosocial correlates of flood impacts in coastal areas of Lagos, Nigeria. In Journal of Environmental Planning and Management 58 (3), pp. 445–463. DOI: 10.1080/09640568.2013.861811#.Ut_3wBD8W01.

**[26] Ajibade, I.; McBean, G. (2014):** Climate extremes and housing rights: A political ecology of impacts, early warning and adaptation constraints in Lagos slum communities. In Geoforum 55, pp. 76–86. DOI: 10.1016/j.geoforum.2014.05.005.

**[27] Ajibade, I.; McBean, G.; Bezner-Kerr, R. (2013):** Urban flooding in Lagos, Nigeria: Patterns of vulnerability and resilience among women. In Global Environmental Change 23 (6), pp. 1714–1725. DOI: 10.1016/j.gloenvcha.2013.08.009.

**[28] Ajibade, I.; Olawuyi, D. S. (2017):** Climate change impacts on housing and property rights in Nigeria and Panama: Toward a rights-based approach to adaptation and mitigation. In D. Stucker, E Lopez-Gunn (Eds.): Adaptation to climate change through water resources management. Capacity, Equity and Sustainability: Routledge, pp. 264–284.

**[29] Akukwe, T. I.; Ogbodo, C. (2015):** Spatial analysis of vulnerability to flooding in Port Harcourt metropolis, Nigeria. In SAGE Open 5 (1), 2158244015575558. DOI: 10.1177/2158244015575558.

**[30] Alou, A. A.; Lutoff, C.; Mounkaila, H. (2019):** Relocalisation préventive suite à la crue de Niamey 2012 : vulnérabilités socio-économiques émergentes et retour en zone inondable. In Cybergeo: European Journal of Geography, Regional and Urban Planning. DOI: 10.4000/cybergeo.32601.

**[31] Amoako, C. (2016):** Brutal presence or convenient absence: The role of the state in the politics of flooding in informal Accra, Ghana. In Geoforum 77, pp. 5–16. DOI: 10.1016/j.geoforum.2016.10.003.

**[32] Amoako, C. (2018):** Emerging grassroots resilience and flood responses in informal settlements in Accra, Ghana. In GeoJournal 83 (5), pp. 949–965. DOI: 10.1007/s10708-017-9807-6.

**[33] Amoako, C.; Cobbinah, P. B.; Mensah Darkwah, R. (2019):** Complex twist of fate: The geopolitics of flood management regimes in Accra, Ghana. In Cities 89, pp. 209–217. DOI: 10.1016/j.cities.2019.02.006.

**[34] Amoako, C.; Inkoom, D. K. B. (2017):** The production of flood vulnerability in Accra, Ghana: Re-thinking flooding and informal urbanisation. In Urban Studies 55 (13), pp. 2903–2922. DOI: 10.1177/0042098016686526.

**[35] Amusat, A. S.; Amusat, K. K. (2013):** Effects of flood on farmers in peri-urban area of Ibadan, Oyo State, Nigeria. In Journal of Environmental Extension 11, pp. 27–31.

**[36] Amuzu, J.; Jallow, B.; Kabo-Bah, A.; Yaffa, S. (2018):** The climate change vulnerability and risk management matrix for the coastal zone of The Gambia. In Hydrology 5 (1), p. 14. DOI: 10.3390/hydrology5010014.

**[37] Antwi, E. K.; Boakye-Danquah, J.; Barima Owusu, A.; Loh, S. K.; Mensah, R.; Boafo, Y. A.; Apronti, P. T. (2015):** Community vulnerability assessment index for flood prone savannah agro-ecological zone: A case study of Wa West District, Ghana. In Weather and Climate Extremes 10, pp. 56–69. DOI: 10.1016/j.wace.2015.10.008.

**[38] Antwi-Boasiako, B. A. (2016):** Insurance and flood risk reduction in Ghana: do insurers penalise homeowners who take precautionary measures? In Environmental Hazards 15 (4), pp. 343–355. DOI: 10.1080/17477891.2016.1209455.

**[39] Antwi-Boasiako, B. A. (2017):** It's beyond my control: The effect of locus of control orientation on disaster insurance adoption. In International Journal of Disaster Risk Reduction 22, pp. 297–303. DOI: 10.1016/j.ijdrr.2017.02.014.

**[40] Atidegla, S. C.; Koumassi, H. D.; Houssou, E. S. (2017):** Variabilité climatique et production maraîchère dans la plaine inondable d’Ahomey-Gblon au Bénin. In International Journal of Biological and Chemical Sciences 11 (5), pp. 2254–2269. DOI: 10.4314/ijbcs.v11i5.24.

**[41] Biconne, R. (2014):** Knowledge sharing on climate change as a resource for adaptation processes: The case of Malika, Senegal. In S. Macchi, M. Tiepolo (Eds.): Climate change vulnerability in southern African cities: Building knowledge for adaptation. Cham: Springer International Publishing, pp. 125–140. Available online at https://doi.org/10.1007/978-3-319-00672-7_8.

**[42] Boamah, S.; Armah, F.; Kuuire, V.; Ajibade, I.; Luginaah, I.; McBean, G. (2015):** Does previous experience of floods stimulate the adoption of coping strategies? Evidence from cross sectional surveys in Nigeria and Tanzania. In Environments 2 (4), pp. 565–585. DOI: 10.3390/environments2040565.

**[43] Bonye, S.; Jasaw, G. (2011):** Traditional coping mechanism in disaster management in the Builsa and Sissala districts of northern Ghana. In European Journal of Social Sciences 25, pp. 204–218.

**[44] Bottazzi, P.; Winkler, M.; Boillat, S.; Diagne, A.; Maman Chabi, Sika, M.; Kpangon, A. et al. (2018):** Measuring subjective flood resilience in suburban Dakar: A before–after evaluation of the “Live with Water” project. In Sustainability 10 (7), p. 2135. DOI: 10.3390/su10072135.

**[45] Bottazzi, P.; Winkler, M. S.; Ifejika Speranza, C. (2019):** Flood governance for resilience in cities: The historical policy transformations in Dakar’s suburbs. In Environmental Science & Policy 93, pp. 172–180. DOI: 10.1016/j.envsci.2018.12.013.

**[46] Boubacar, S.; Pelling, M.; Barcena, A.; Montandon, R. (2017):** The erosive effects of small disasters on household absorptive capacity in Niamey: a nested HEA approach. In Environment and Urbanization 29 (1), pp. 33–50. DOI: 10.1177/0956247816685515.

**[47] Brisibe, W. G.; Pepple, T. D. (2018):** Lessons learnt from the 2012 flood disaster: Implications for post-flood building design and construction in Yenagoa, Nigeria. In Civil Engineering and Architecture 6 (3), pp. 171–180. DOI: 10.13189/cea.2018.060307.

**[48] Campion, B. B.; Venzke, J.-F. (2013):** Rainfall variability, floods and adaptations of the urban poor to flooding in Kumasi, Ghana. In Natural Hazards 65 (3), pp. 1895–1911. DOI: 10.1007/s11069-012-0452-6.

**[49] Chukwu, M. N. (2015):** Impact of flooding on fishermen’s families in Pedro community, Iwaya-Lagos, Nigeria. In Journal of Applied Sciences and Environmental Management 18 (4), pp. 647–651. DOI: 10.4314/jasem.v18i4.13.

**[50] Chukwuma, O. M.; Uchenna, O. F. (2018):** A comparative analysis of flooding in Warri and Port Harcourt urban areas of the Niger Delta region in southern Nigeria. In Arabian Journal of Geosciences 11 (8), p. 166. DOI: 10.1007/s12517-018-3525-3.

**[51] Cirella, G.; Iyalomhe, F.; Adekola, O. (2019):** Determinants of flooding and strategies for mitigation: Two-year case study of Benin City. In Geosciences (Switzerland) 9. DOI: 10.3390/geosciences9030136.

**[52] Cissé, O.; Sèye, M. (2016):** Flooding in the suburbs of Dakar: impacts on the assets and adaptation strategies of households or communities. In Environment and Urbanization 28 (1), pp. 183–204. DOI: 10.1177/0956247815613693.

**[53] Clark-Ginsberg, A. (2017):** Participatory risk network analysis: A tool for disaster reduction practitioners. In International Journal of Disaster Risk Reduction 21, pp. 430–437. DOI: 10.1016/j.ijdrr.2017.01.006.

**[54] Codjoe, S. N. A.; Atidoh, L. K.; Burkett, V. (2012):** Gender and occupational perspectives on adaptation to climate extremes in the Afram Plains of Ghana. In Climatic Change 110 (1), pp. 431–454. DOI: 10.1007/s10584-011-0237-z.

**[55] Codjoe, S. N. A.; Issah, A. D. (2016):** Cultural dimension and adaptation to floods in a coastal settlement and a savannah community in Ghana. In GeoJournal 81 (4), pp. 615–624. DOI: 10.1007/s10708-015-9641-7.

**[56] Codjoe, S. N. A.; Nyamedor, F. H.; Sward, J.; Dovie, D. B. (2017):** Environmental hazard and migration intentions in a coastal area in Ghana: a case of sea flooding. In Population and Environment 39 (2), pp. 128–146. DOI: 10.1007/s11111-017-0284-0.

**[57] Codjoe, S. N. A.; Owusu, G.; Burkett, V. (2014):** Perception, experience, and indigenous knowledge of climate change and variability: the case of Accra, a sub-saharan African city. In Regional Environmental Change 14 (1), pp. 369–383. DOI: 10.1007/s10113-013-0500-0.

**[58] Coker, A. A.; Adebayo, C. O.; Odoemena, B. C.; Akogun, E. O.; Ezinne, C. G. (2014):** Flood and cassave productivity in Kogi State, Nigeria: A quantitative analysis using cross-sectional data. In Ethiopian Journal of Environmental Studies and Management 7 (6), pp. 599–608. DOI: 10.4314/ejesm.v7i6.2.

**[59] Danso, S. Y.; Addo, I. Y. (2017):** Coping strategies of households affected by flooding: A case study of Sekondi-Takoradi Metropolis in Ghana. In Urban Water Journal 14 (5), pp. 539–545. DOI: 10.1080/1573062X.2016.1176223.

**[60] Derbile, E.; File, D.; Dongzagla, A. (2016):** The double tragedy of agriculture vulnerability to climate variability in Africa: How vulnerable is smallholder agriculture to rainfall variability in Ghana? In Jàmbá: Journal of Disaster Risk Studies 8. DOI: 10.4102/jamba.v8i3.249.

**[61] Diagne, K. (2007):** Governance and natural disasters: addressing flooding in Saint Louis, Senegal. In Environment and Urbanization 19 (2), pp. 552–562. DOI: 10.1177/0956247807082836.

**[62] Diagne, K.; Ndiaye, A. (2012):** History, governance and the millennium development goals: Flood risk reduction in Saint-Louis, Senegal. In M. Pelling, B. Wisner (Eds.): Disaster risk reduction. Cases from Urban Africa: Routledge, pp. 147–167.

**[63] Egbinola, C. N.; Olaniran, H. D.; Amanambu, A. C. (2017):** Flood management in cities of developing countries: the example of Ibadan, Nigeria. In Journal of Flood Risk Management 10 (4), pp. 546–554. DOI: 10.1111/jfr3.12157.

**[64] Ekpo, F.; Nzegblue, E. C. (2012):** Climate change impact and adaptation opportunities on agricultural production in communities around Itu bridge-head in Itu LGA, Akwa Ibom State, Nigeria. In Agris On-line Papers in Economics and Informatics 2, pp. 2239–2250. DOI: 10.6088/ijes.002020300107.

**[65] Enete, A.; Nneamaka, O.; Ozor, N.; Lilian, M. (2016):** Socioeconomic assessment of flooding among farm households in Anambra state, Nigeria. In International Journal of Climate Change Strategies and Management 8, pp. 96–111. DOI: 10.1108/IJCCSM-07-2014-0084.

**[66] Evadzi, P. I. K.; Scheffran, J.; Zorita, E.; Hünicke, B. (2018):** Awareness of sea-level response under climate change on the coast of Ghana. In Journal of Coastal Conservation 22 (1), pp. 183–197. DOI: 10.1007/s11852-017-0569-6.

**[67] Ezemonye, M. N.; Emeribe, C. N. (2014):** Flooding and household preparedness in Benin City, Nigeria. In Mediterranean Journal of Social Sciences. DOI: 10.5901/mjss.2014.v5n1p547.

**[68] Frick-Trzebitzky, F. (2017):** Crafting adaptive capacity: Institutional bricolage in adaptation to urban flooding in Greater Accra. In Water Alternatives 10 (2), pp. 625–647.

**[69] Frick-Trzebitzky, F.; Bruns, A. (2019):** Disparities in the implementation gap: adaptation to flood risk in the Densu Delta, Accra, Ghana. In Journal of Environmental Policy & Planning 21 (5), pp. 577–592. DOI: 10.1080/1523908X.2017.1343136.

**[70] Glago, F. J. (2019):** Household disaster awareness and preparedness: A case study of flood hazards in Asamankese in the West Akim Municipality of Ghana. In Jàmbá: Journal of Disaster Risk Studies 11 (1), p. 789. DOI: 10.4102/jamba.v11i1.789.

**[71] Gobo A.E.; Abam T.K.S. (1991):** The 1988 floods in the Niger Delta: The Case of Ndoni. In Journal of Meteorology 16 (163), pp. 293–299.

**[72] Gobo A.E.; Abam T.K.S.; Ogam F.N. (2006):** The application of Kruskal‐Wallis technique for flood prediction in the Niger Delta, Nigeria. In Management of Environmental Quality: An International Journal 17 (3), pp. 275–288. DOI: 10.1108/14777830610658692.

**[73] Goyol, S.; Pathirage, C. (2018):** Farmers perceptions of climate change related events in Shendam and Riyom, Nigeria. In Economies 6, p. 70. DOI: 10.3390/economies6040070.

**[74] Hetcheli, F. (2013):** Risques pluviometriques et nouvelles orientations des agriculteurs du canton de Togblekope (Basse Vallee de Zio) au Togo. In Journal de la Recherche Scientifique de l’Université de Lomé - Série B 15 (2), pp. 135–149.

**[75] Ibitoye, M. O.; Komolafe, A. A.; Adegboyega, A. S.; Adebola, A. O.; Oladeji, O. D. (2019):** Analysis of vulnerable urban properties within river Ala floodplain in Akure, Southwestern Nigeria. In Spatial Information Research. DOI: 10.1007/s41324-019-00298-6.

**[76] Ibrahim, A. H.; Abdullahi, S. Z. (2016):** Flood menace in Kaduna metropolis: Impacts, remedial and management strategies. In Science World Journal 11 (2), 16-22.

**[77] Ingram, K.T; Roncoli, M.C; Kirshen, P.H (2002):** Opportunities and constraints for farmers of West Africa to use seasonal precipitation forecasts with Burkina Faso as a case study. In Agricultural Systems 74 (3), pp. 331–349. DOI: 10.1016/S0308-521X(02)00044-6.

**[78] Jallow, B. P.; Toure, S.; Barrow, M. M. K.; Mathieu, A. A. (1999):** Coastal zone of The Gambia and the Abidjan region in Côte d'Ivoire. Sea level rise vulnerability, response strategies, and adaptation options. In Climate Research 12 (2/3), pp. 129–136.

**[79] Kablan, M.; Dongo, K.; Coulibaly, M. (2017):** Assessment of social vulnerability to flood in urban Côte d’Ivoire using the MOVE framework. In Water 9, p. 292. DOI: 10.3390/w9040292.

**[80] Kablan, M.; Dongo, K.; Fokou, G.; Coulibaly, M. (2019):** Assessing population perception and socioeconomic impact related to flood episodes in urban Côte d’Ivoire. In International Journal of Biological and Chemical Sciences 13 (4), pp. 2210–2223. DOI: 10.4314/ijbcs.v13i4.26.

**[81] Kielland, A. (2016):** The role of risk perception in child mobility decisions in West Africa, empirical evidence from Benin. In World Development 83, pp. 312–324. DOI: 10.1016/j.worlddev.2016.01.008.

**[82] Kloos, J.; Renaud, F. (2014):** Organic cotton production as an adaptation option in north-west Benin. In Outlook on Agriculture 43, pp. 91–100. DOI: 10.5367/oa.2014.0166.

**[83] Komi, K.; Amisigo, B.; Diekkrüger, B. (2016):** Integrated flood risk assessment of rural communities in the Oti River basin, West Africa. In Hydrology 3 (4), p. 42. DOI: 10.3390/hydrology3040042.

**[84] Lamond, J.; Adekola, O.; Adelekan, I.; Eze, E.; Ujoh, F. (2019):** Information for adaptation and response to flooding, multi-stakeholder perspectives in Nigeria. In Climate 7, pp. 1–18. DOI: 10.3390/cli7040046.

**[85] Leclercq, R. (2017):** The politics of risk policies in Dakar, Senegal. In International Journal of Disaster Risk Reduction 26, pp. 93–100. DOI: 10.1016/j.ijdrr.2017.09.031.

**[86] Lokonon, B. O. K. (2016):** Urban households' attitude towards flood risk, and waste disposal: Evidence from Cotonou. In International Journal of Disaster Risk Reduction 19, pp. 29–35. DOI: 10.1016/j.ijdrr.2016.08.015.

**[87] Lolig, V.; Donkoh, S.; Obeng, F. K.; Gershon, I.; Ansah, I.; Jasaw, G. et al. (2014):** Households' coping strategies in drought-and flood-prone communities in Northern Ghana. In Journal of Disaster Research 9, pp. 542–553.

**[88] Maheu, A. (2012):** Urbanization and flood vulnerability in a peri-urban neighbourhood of Dakar, Senegal: How can participatory GIS contribute to flood management? In W. Leal Filho (Ed.): Climate change and the sustainable use of water resources. Berlin, Heidelberg: Springer Berlin Heidelberg, pp. 185–207. Available online at https://doi.org/10.1007/978-3-642-22266-5_12.

**[89] Markantonis, V.; Farinosi, F.; Dondeynaz, C.; Ameztoy, I.; Pastori, M.; Marletta, L. et al. (2018):** Assessing floods and droughts in the Mékrou River basin (West Africa): A combined household survey and climatic trends analysis approach. In Natural Hazards and Earth System Sciences 18 (4), pp. 1279–1296. DOI: 10.5194/nhess-18-1279-2018.

**[90] Martins, B.; Nunes, A.; Lourenço, L.; Velez-Castro, F. (2019):** Flash Flood Risk Perception by the Population of Mindelo, S. Vicente (Cape Verde). In Water 11 (9). DOI: 10.3390/w11091895.

**[91] Mbow, C.; Diop, A.; Diaw, A. T.; Niang, C. I. (2008):** Urban sprawl development and flooding at Yeumbeul suburb (Dakar-Senegal). In African Journal of Environmental Science and Technology 2 (4), pp. 75–88.

**[92] Milliano, C.W.J. de (2015):** Luctor et emergo, exploring contextual variance in factors that enable adolescent resilience to flooding. In International Journal of Disaster Risk Reduction 14, pp. 168–178. DOI: 10.1016/j.ijdrr.2015.07.005.

**[93] Morand, P.; Kodio, A.; Andrew, N.; Sinaba, F.; Lemoalle, J.; Béné, C. (2012):** Vulnerability and adaptation of African rural populations to hydro-climate change: experience from fishing communities in the Inner Niger Delta (Mali). In Climatic Change 115 (3), pp. 463–483. DOI: 10.1007/s10584-012-0492-7.

**[94] Ndamani, F.; Watanabe, T. (2016):** Determinants of farmers’ adaptation to climate change: A micro level analysis in Ghana. In Scientia Agricola 73, pp. 201–208. DOI: 10.1590/0103-9016-2015-0163.

**[95] Ngwese, N. M.; Saito, O.; Sato, A.; Agyeman Boafo, Y.; Jasaw, G. (2018):** Traditional and local knowledge practices for disaster risk reduction in Northern Ghana. In Sustainability 10 (3), p. 825. DOI: 10.3390/su10030825.

**[96] Ntajal, J.; Lamptey, B. L.; Mahamadou, I. B.; Nyarko, B. K. (2017):** Flood disaster risk mapping in the Lower Mono River Basin in Togo, West Africa. In International Journal of Disaster Risk Reduction 23, pp. 93–103. DOI: 10.1016/j.ijdrr.2017.03.015.

**[97] Nti, Frank; Barkley, Andrew (2013):** The impact of human capital on the response to climate change vulnerability among farm families in Northern Ghana. In Journal of International Agricultural Trade and Development 8.

**[98] Nyantakyi-Frimpong, H. (2019):** Unmasking difference: intersectionality and smallholder farmers’ vulnerability to climate extremes in Northern Ghana. In Gender, Place & Culture, pp. 1–19. DOI: 10.1080/0966369X.2019.1693344.

**[99] Odemerho, F. O. (2014):** Building climate change resilience through bottom-up adaptation to flood risk in Warri, Nigeria. In Environment and Urbanization 27 (1), pp. 139–160. DOI: 10.1177/0956247814558194.

**[100] Odjugo, P. A. O. (2012):** Valuing the cost of environmental degradation in the face of changing climate: Emphasis on flood and erosion in Benin City, Nigeria. In African Journal of Environmental Science and Technology 6 (1), pp. 17–27. DOI: 10.5897/AJEST11.174.

**[101] Odubo, T. (2014):** The socio-cultural effects of flooding in Bayelsa State: A case study of Southern Ijaw Local Government Area. In Mediterranean Journal of Social Sciences 5, pp. 1443–1450. DOI: 10.5901/mjss.2014.v5n27p1443.

**[102] Olanrewaju, C. C.; Chitakira, M.; Olanrewaju, O. A.; Louw, E. (2019):** Impacts of flood disasters in Nigeria: A critical evaluation of health implications and management. In Jàmbá: Journal of Disaster Risk Studies 11 (1), p. 557. DOI: 10.4102/jamba.v11i1.557.

**[103] Ologunorisa, T. E.; Adeyemo, A. (2005):** Public perception of flood hazard in the Niger Delta, Nigeria. In Environmentalist 25 (1), pp. 39–45. DOI: 10.1007/s10669-005-3095-2.

**[104] Olokesusi, F.; Olorunfemi, F. B.; Onwuemele, A.; Oke, M. O. (2015):** Awareness of and responses to the 2011 flood warnings among vulnerable communities in Lagos, Nigeria. In B. Werlen (Ed.): Global sustainability, vol. 27. Cham: Springer International Publishing, pp. 203–223.

**[105] Onu, B.; Price, T.; Surendran, S.; Timbiri, A. (2013):** Peoples’ perception on the effects of floods in the riverine areas of Ogbia Local Government Area of Bayelsa State, Nigeria. In Knowledge Management: An International Journal 12, pp. 22–43. DOI: 10.18848/2327-7998/CGP/v12i02/50793.

**[106] Onwuemele, A. (2012):** Cities in the flood: Vulnerability and disaster risk management: Evidence from Ibadan, Nigeria. In W. G. Holt (Ed.): Urban areas and global climate change, vol. 12: Emerald Group Publishing Limited (Research in Urban Sociology), pp. 277–299.

**[107] Onwuemele, A. (2018):** Public perception of flood risks and disaster preparedness in Lagos megacity, Nigeria. In Academic Journal of Interdisciplinary Studies 7 (3), pp. 179–185. DOI: 10.2478/ajis-2018-0068.

**[108] Osayomi, T.; Oladosu, O. S. (2016):** “Expect more floods in 2013”: An analysis of flood preparedness in the flood prone city of Ibadan, Nigeria. In African Journal of Sustainable Development 6 (2), pp. 215–237.

**[109] Osman, A.; Nyarko, B. K.; Mariwah, S. (2016):** Vulnerability and risk levels of communities within Ankobra estuary of Ghana. In International Journal of Disaster Risk Reduction 19, pp. 133–144. DOI: 10.1016/j.ijdrr.2016.08.016.

**[110] Ottah, G. A. (2017):** Impact of Radio Kogi’s flood disaster awareness campaign on residents of Ibaji Local Government Area of Kogi State, Nigeria. In International Journal of Arts and Humanities (IJAH) Ethiopia 6 (3), pp. 80–97. DOI: 10.4314/ijah.v6i3.7.

**[111] Owusu-Ansah, J. K.; Dery, J. M.; Amoako, C. (2019):** Flood vulnerability and coping mechanisms around the Weija Dam near Accra, Ghana. In GeoJournal 84 (6), pp. 1597–1615. DOI: 10.1007/s10708-018-9939-3.

**[112] Oyekale, A. S. (2013):** Fishing folks' access to early warning and post flood assistances in Lagos State, Nigeria: Application of seemingly unrelated bivariate probit (SUBP) regression. In Journal of Animal and Veterinary Advances 12, pp. 607–611. DOI: 10.3923/javaa.2013.607.611.

**[113] Oyekale, A. S.; Oladele, O. I.; Mukela, F. (2013):** Impacts of flooding on coastal fishing folks and risk adaptation behaviours in Epe, Lagos State. In African Journal of Agricultural Research 8 (26), pp. 3392–3405. DOI: 10.5897/AJAR12.730.

**[114] Oyerinde, G. T.; Lawin, E. A.; Odofin, A. J. (2017):** Farmers’ responses to changing hydrological trends in the Niger Basin parts of Benin. In Hydrology 4 (4), p. 52. DOI: 10.3390/hydrology4040052.

**[115] Redshaw, P.; Boon, D.; Campbell, G.; Willis, M.; Mattai, J.; Free, M. et al. (2019):** The 2017 Regent Landslide, Freetown Peninsula, Sierra Leone. In Quarterly Journal of Engineering Geology and Hydrogeology 52 (4), pp. 435–444. DOI: 10.1144/qjegh2018-187.

**[116] Sabino, A. A.; Querido, A. L.; Sousa, M. I. (1999):** Flood management in Cape Verde. The case study of Praia. In Urban Water 1 (2), pp. 161–166. DOI: 10.1016/S1462-0758(00)00011-X.

**[117] Saidu, I. (2009):** An analysis of Loko flood disaster resettlement scheme, in Song Local Government Area of Adamawa State, Nigeria. In FUTY Journal of the Environment 4 (1), pp. 19–27.

**[118] Salami, R.; Meding, J. v.; Giggins, H. (2017):** Vulnerability of human settlements to flood risk in the core area of Ibadan metropolis, Nigeria. In Jàmbá: Journal of Disaster Risk Studies 9, a371. DOI: 10.4102/jamba.v9i1.371.

**[119] Samaddar, S.; Yokomatsu, M.; Dzivenu, T.; Oteng-Ababio, M.; Adams, M. R.; Dayour, F.; Ishikawa, H. (2014):** Assessing rural communities concerns for improved climate change adaptation strategies in Northern Ghana. In Journal of Disaster Research 9 (4), pp. 529–541. DOI: 10.20965/jdr.2014.p0529.

**[120] Schaer, C. (2015):** Condemned to live with one’s feet in water? In International Journal of Climate Change Strategies and Management 7 (4), pp. 534–551. DOI: 10.1108/IJCCSM-03-2014-0038.

**[121] Schaer, C.; Hanonou, E. K. (2017):** The real governance of disaster risk management in peri-urban Senegal: Delivering flood response services through co-production. In Progress in Development Studies 17 (1), pp. 38–53. DOI: 10.1177/1464993416674301.

**[122] Schaer, C.; Thiam, M. D.; Nygaard, I. (2018):** Flood management in urban Senegal: an actor-oriented perspective on national and transnational adaptation interventions. In Climate and Development 10 (3), pp. 243–258. DOI: 10.1080/17565529.2017.1291405.

**[123] Schlef, K.; Kaboré, L.; Karambiri, H.; Yang, Y.-C.; Brown, C. (2018):** Relating perceptions of flood risk and coping ability to mitigation behavior in West Africa: Case study of Burkina Faso. In Environmental Science & Policy 89, pp. 254–265. DOI: 10.1016/j.envsci.2018.07.013.

**[124] Schultz, K.; Adler, L. (2017):** Addressing climate change impacts in the Sahel using vulnerability reduction credits. In M. Tiepolo, A. Pezzoli, V. Tarchiani (Eds.): Renewing local planning to face climate change in the tropics. Cham: Springer International Publishing, pp. 343–363.

**[125] Serpantié, G.; Dorée, A.; Fusillier, J.-L.; Moity-Maizi, P.; Lidon, B.; Douanio, M. et al. (2019):** Nouveaux risques dans les bas-fonds des terroirs soudaniens. Une étude de cas au Burkina Faso. In Cahiers Agricultures 28, pp. 1–10. DOI: 10.1051/cagri/2019020.

**[126] Soneye, A. (2014):** An overview of humanitarian relief supply chains for victims of perennial flood disasters in Lagos, Nigeria (2010-2012). In Journal of Humanitarian Logistics and Supply Chain Management 4 (2), pp. 179–197. DOI: 10.1108/JHLSCM-01-2014-0004.

**[127] Sousa, J.; Luz, A. L. (2018):** ‘The tides rhyme with the Moon’: The impacts of knowledge transmission and strong spring tides on rice farming in Guinea-Bissau. In Human Ecology 46 (2), pp. 147–157. DOI: 10.1007/s10745-018-9980-3.

**[128] Spaling, H. (2003):** Innovation in environmental assessment of community-based projects in sub-Saharan Africa. In The Canadian Geographer/Le Géographe canadien 47 (2), pp. 151–168. DOI: 10.1111/1541-0064.00007.

**[129] Tasantab, J. C. (2019):** Beyond the plan: How land use control practices influence flood risk in Sekondi-Takoradi. In Jàmbá: Journal of Disaster Risk Studies 11 (1), p. 638. DOI: 10.4102/jamba.v11i1.638.

**[130] Tiepolo, M.; Rosso, M.; Massazza, G.; Belcore, E.; Issa, S.; Braccio, S. (2019):** Flood assessment for risk-informed planning along the Sirba River, Niger. In Sustainability 11, pp. 1–18. DOI: 10.3390/su11154003.

**[131] Tschakert, P.; Sagoe, R.; Ofori-Darko, G.; Codjoe, S. N. A. (2010):** Floods in the Sahel: an analysis of anomalies, memory, and anticipatory learning. In Climatic Change 103 (3), pp. 471–502. DOI: 10.1007/s10584-009-9776-y.

**[132] Twum, K. O.; Abubakari, M. (2019):** Cities and floods: A pragmatic insight into the determinants of households' coping strategies to floods in informal Accra, Ghana. In Jàmbá: Journal of Disaster Risk Studies 11 (1), p. 608. DOI: 10.4102/jamba.v11i1.608.

**[133] Vedeld, T.; Coly, A.; Ndour, N. M.; Hellevik, S. (2016):** Climate adaptation at what scale? Multi-level governance, resilience, and coproduction in Saint Louis, Senegal. In Natural Hazards 82 (2), pp. 173–199. DOI: 10.1007/s11069-015-1875-7.

**[134] Vissin, E. W.; Hedible, S.; Amoussou, E.; Totin, H. S.; Odoulami, L.; Etene, C. et al. (2016):** Variabilité climatique et hydrologique dans la basse vallée de l'Ouémé à Bonou. In Journal de la Recherche Scientifique de l’Université de Lomé - Série B 18 (2), pp. 69–81.

**[135] Wahab, B.; Falola, O. (2017):** The consequences and policy implications of urban encroachment into flood-risk areas: the case of Ibadan. In Environmental Hazards 16 (1), pp. 1–20. DOI: 10.1080/17477891.2016.1211505.

**[136] Yankson, P. W. K.; Owusu, A. B.; Owusu, G.; Boakye-Danquah, J.; Tetteh, J. D. (2017):** Assessment of coastal communities’ vulnerability to floods using indicator-based approach: a case study of Greater Accra Metropolitan Area, Ghana. In Natural Hazards 89 (2), pp. 661–689. DOI: 10.1007/s11069-017-2985-1.

**[137] Yawson, D. O.; Adu, M. O.; Armah, F. A.; Kusi, J.; Ansah, I. G.; Chiroro, C. (2015):** A needs-based approach for exploring vulnerability and response to disaster risk in rural communities in low income countries. In Australasian Journal of Disaster and Trauma Studies 19, pp. 27–36.

**[138] Young, H. R.; Cornforth, R. J.; Gaye, A. T.; Boyd, E. (2019):** Event attribution science in adaptation decision-making: the context of extreme rainfall in urban Senegal. In Climate and Development 11 (9), pp. 812–824. DOI: 10.1080/17565529.2019.1571401.

**Annex 3** Number of selected documents by year of publication (from 1991 to 2019)

**Annex 4** Research areas by country (multiple mentions possible in each document)

**Annex 5** Type of geographical area the research areas are located in (multiple mentions possible per case study)

**Annex 6** Types of flood mentioned in selected documents (multiple mentions possible in each document)

**Annex 7** Types of methods used for primary data collection (multiple mentions possible in each document)

**Annex 8** Summary of FRM measures before the onset of the most recent flood event grouped by categories

**Infrastructural** = *all measures that describe an infrastructural intervention to mitigate the hazard or to overcome its adverse impacts*

**Mutual support** = *all measures that summarize mutual support to reduce the risk of and overcome the adverse impacts of floods between people based on solidarity*

**Maintenance activities** = a*ll measures that aim at maintaining infrastructure, tools or performing clean up activities to better reduce the risk of or overcome the adverse impacts of floods*

**Awareness-raising, training & education** = *all measures that aim at raising awareness of, providing training, and education on relevant topics to people at risk to reduce the risk of or to overcome the adverse impacts of floods*

**Information resources** = *all measures that harness information channels and platforms to reduce flood risk or to overcome the adverse impacts of floods*

**Preparing/providing assistance & response** = *all measures that aim at preparing the provision of assistance or relief to reduce flood risk or at providing it to overcome the adverse impacts of floods*

**Relocation** = *all measures that aim at reducing flood risk or overcoming the adverse impacts of floods through the movement of people or assets at risk out of the flood zone*

**Spatial planning interventions** = *All measures that aim at reducing flood risk by the application of spatial planning interventions or by the new creation of such*

**Local knowledge and skills** = *the explicit consideration of place-specific knowledge of risk, possibilities to reduce it and to overcome its adverse effects*

**Policies** = *All measures from the* *sphere of policies by which flood risk is intended to be reduced or adverse flood effects are attempted to be overcome*

**Insurance** = a *formalized risk transfer arrangement with an insurance company as the risk carrier*

**Research & assessment** = *all measures that aim at generating information or knowledge to reduce flood risk or to alleviate the adverse effects of a flood event*

**Nature-based solutions** = *the use of ecosystem services to reduce flood risk or to alleviate the adverse impacts of a flood event*

**Modification of practices** = *the modification of previously applied practices to reduce flood risk or to alleviate the adverse impacts of a flood event*

**Risk retention** = *retaining of resources to alleviate the adverse effects of unaddressed risk in case of flood event*

**Modification of livelihood** = *the modification of previously practiced livelihoods to reduce flood risk or to alleviate the adverse impacts of a flood event*

**Religious & spiritual activities** = *the use of religious or spiritual activities to perceive flood risk or to alleviate the adverse impacts of a flood event*

**Health care** = *the use of health care to reduce flood risk or to alleviate the adverse impacts of a flood event*

**Annex 9** Impacts from residual flood risks from the most recent flood event

**Material damage** = *Damage of physical assets and resources used for livelihoods*

**Health** = *Adverse health impacts of the flood event*

**Economic losses** = *Adverse effects on the economic situation of the population at risk and their cost of covering it*

**Environmental degradation** = *Adverse flood impacts which lead to a degradation or pollution of the environment*

**Lack of food/drinking water** = *Adverse flood impacts leading to a scarcity of food or drinking water*

**Displacement & homelessness** = *Adverse flood impacts leading to a displacement from or a loss of the residence of the people at risk*

**Interruption of social activities** = *Adverse flood impacts disturbing common social activities*

**Lack of mobility** = *Adverse flood impacts impairing the ability of people at risk to move*

**Annex 10** Measures to address impacts from residual flood risks after the onset of the most recent flood event

**Infrastructural** = *all measures that describe an infrastructural intervention to mitigate the hazard or to overcome its adverse impacts*

**Relocation** = *all measures that aim at reducing flood risk or overcoming the adverse impacts of floods through the movement of people or assets at risk out of the flood zone*

**Mutual support** = *all measures that summarize mutual support to reduce the risk of and overcome the adverse impacts of floods between people based on solidarity*

**Providing assistance & response** = *all measures that aim at providing assistance or relief to overcome the adverse impacts of floods*

**Modification of practices** = *the modification of previously applied practices to reduce flood risk or to alleviate the adverse impacts of a flood event*

**Maintenance activities** = *all measures that aim at maintaining infrastructure, tools or performing clean up activities to better reduce the risk of or overcome the adverse impacts of floods*

**Using retained resources** = using *retained resources to alleviate the adverse effects of unaddressed risk in case of a flood event*

**Modification of livelihood** = *the modification of previously practiced livelihoods to reduce flood risk or to alleviate the adverse impacts of a flood event*

**Local knowledge & skills** = *the explicit consideration of place-specific knowledge of risk, possibilities to reduce it and to overcome its adverse effects*

**Health care** = *the use of health care to reduce flood risk or to alleviate the adverse impacts of a flood event*

**Nature-based solutions** = *the use of ecosystem services to reduce flood risk or to alleviate the adverse impacts of a flood event*

**Religious & spiritual activities** = *the use of religious or spiritual activities to perceive flood risk or to alleviate the adverse impacts of a flood event*

**Research & assessment** = *all measures that aim at generating information or knowledge to reduce flood risk or to alleviate the adverse effects of a flood event*

**Awareness-raising, training and education** = *all measures that aim at raising awareness of, providing training, and education on relevant topics to people at risk to reduce the risk of or to overcome the adverse impacts of floods*

**Spatial planning interventions** = *All measures that aim at reducing flood risk by the application of spatial planning interventions or by the new creation of such*

**Information resources** = *all measures that harness information channels and platforms to reduce flood risk or to overcome the adverse impacts of floods*

**Policies** = *all measures from the* *sphere of policies by which flood risk is intended to be reduced or adverse flood effects are attempted to be overcome*

**Insurance** = a *formalized risk transfer arrangement with an insurance company as the risk carrier*

**Annex 11** Recommendations in selected case studies to further reduce residual flood risk

**Policies** = *All measures from the* *sphere of policies by which flood risk is intended to be reduced or adverse flood effects are attempted to be overcome*

**Awareness-raising, training and education** = *all measures that aim at raising awareness of, providing training, and education on relevant topics to people at risk to reduce the risk of or to overcome the adverse impacts of floods*

**Infrastructural** = *all measures that describe an infrastructural intervention to mitigate the hazard or to overcome its adverse impacts*

**Spatial planning interventions** = *All measures that aim at reducing flood risk by the application of spatial planning interventions or by the new creation of such*

**Research & assessment** = *all measures that aim at generating information or knowledge to reduce flood risk or to alleviate the adverse effects of a flood event*

**Information resources** = *all measures that harness information channels and platforms to reduce flood risk or to overcome the adverse impacts of floods*

**(Preparing/providing) assistance & response** = *all measures that aim at preparing the provision of assistance or relief to reduce flood risk or at providing it to overcome the adverse impacts of floods*

**Relocation** = *all measures that aim at reducing flood risk or overcoming the adverse impacts of floods through the movement of people or assets at risk out of the flood zone*

**Maintenance activities** = *all measures that aim at maintaining infrastructure, tools or performing clean up activities to better reduce the risk of or overcome the adverse impacts of floods*

**Insurance** = *a formalized risk transfer arrangement with an insurance company as the risk carrier*

**Modification of practices** = *the modification of previously applied practices to reduce flood risk or to alleviate the adverse impacts of a flood event*

**Nature-based solutions** = *the use of ecosystem services to reduce flood risk or to alleviate the adverse impacts of a flood event*

**Local knowledge & skills** = *the explicit consideration of place-specific knowledge of risk, possibilities to reduce it and to overcome its adverse effects*

**Mutual support** = *all measures that summarize mutual support to reduce the risk of and overcome the adverse impacts of floods between people based on solidarity*

**Health care** = *the use of health care to reduce flood risk or to alleviate the adverse impacts of a flood event*

**Modification of livelihood** = *the modification of previously practiced livelihoods to reduce flood risk or to alleviate the adverse impacts of a flood event*

**Risk retention** = *retaining of resources to alleviate the adverse effects of unaddressed risk in case of flood event*

**Religious & spiritual activities** = *the use of religious or spiritual activities to perceive flood risk or to alleviate the adverse impacts of a flood event*
